# The Novel Gene *CRNDE* Encodes a Nuclear Peptide (CRNDEP) Which Is Overexpressed in Highly Proliferating Tissues

**DOI:** 10.1371/journal.pone.0127475

**Published:** 2015-05-15

**Authors:** Lukasz Michal Szafron, Anna Balcerak, Ewa Anna Grzybowska, Barbara Pienkowska-Grela, Anna Felisiak-Golabek, Agnieszka Podgorska, Magdalena Kulesza, Natalia Nowak, Pawel Pomorski, Juliusz Wysocki, Tymon Rubel, Agnieszka Dansonka-Mieszkowska, Bozena Konopka, Martyna Lukasik, Jolanta Kupryjanczyk

**Affiliations:** 1 Department of Pathology and Laboratory Diagnostics, Maria Sklodowska-Curie Memorial Cancer Center and Institute of Oncology, Warsaw, Poland; 2 Department of Molecular and Translational Oncology, Maria Sklodowska-Curie Memorial Cancer Center and Institute of Oncology, Warsaw, Poland; 3 Neurobiology Center, Laboratory of Imaging Tissue Structure and Function, Nencki Institute of Experimental Biology, Warsaw, Poland; 4 Multimodal Laboratory of Cell Adhesion and Motility, NanoBioGeo Consortium, Nencki Institute of Experimental Biology, Warsaw, Poland; 5 Department of Biochemistry, Laboratory Of Molecular Basis of Cell Motility, Nencki Institute of Experimental Biology, Warsaw, Poland; 6 The Institute of Radioelectronics, Warsaw University of Technology, Warsaw, Poland; Sudbury Regional Hospital, CANADA

## Abstract

*CRNDE*, recently described as the lncRNA-coding gene, is overexpressed at RNA level in human malignancies. Its role in gametogenesis, cellular differentiation and pluripotency has been suggested as well. Herein, we aimed to verify our hypothesis that the *CRNDE* gene may encode a protein product, CRNDEP. By using bioinformatics methods, we identified the 84-amino acid ORF encoded by one of two *CRNDE *transcripts, previously described by our research team. This ORF was cloned into two expression vectors, subsequently utilized in localization studies in HeLa cells. We also developed a polyclonal antibody against CRNDEP. Its specificity was confirmed in immunohistochemical, cellular localization, Western blot and immunoprecipitation experiments, as well as by showing a statistically significant decrease of endogenous CRNDEP expression in the cells with transient shRNA-mediated knockdown of *CRNDE*. Endogenous CRNDEP localizes predominantly to the nucleus and its expression seems to be elevated in highly proliferating tissues, like the parabasal layer of the squamous epithelium, intestinal crypts or spermatocytes. After its artificial overexpression in HeLa cells, in a fusion with either the EGFP or DsRed Monomer fluorescent tag, CRNDEP seems to stimulate the formation of stress granules and localize to them. Although the exact role of CRNDEP is unknown, our preliminary results suggest that it may be involved in the regulation of the cell proliferation. Possibly, CRNDEP also participates in oxygen metabolism, considering our *in silico* results, and the correlation between its enforced overexpression and the formation of stress granules. This is the first report showing the existence of a peptide encoded by the *CRNDE* gene.

## Introduction

The *CRNDE* (colorectal neoplasia differentially expressed, formerly known as *LOC388279* or *LOC643911*) gene is localized to the long arm of chromosome 16 (16q12.2) in human. According to the current state of knowledge, *CRNDE* is classified as the lncRNA-coding gene [1]. Its RNA products were shown to be overexpressed in colorectal carcinomas, gliomas and other solid tumors and leukemias [2, 3]. We have identified *CRNDE* as the gene with the second highest fold change value (FC = 5.7) among genes negatively affecting prognosis in a group of ovarian cancer patients treated with taxane/platinum (TP) regimens [4, 5]. The *CRNDE* gene is also thought to be implicated in neuronal differentiation, gametogenesis and other developmental processes [6]. Its mouse *ortholog*, named *linc1399*, is thought to be associated with the maintenance of cellular pluripotency [6, 7]. Likely, *CRNDE* transcripts are involved in epigenetic regulation of gene expression, since Khalil et al. [8] showed that they may interact with chromatin-modifying complexes. This interaction affects expression of genes significantly overlapping with those controlled by the polycomb repressive complex 2 (PRC2).

There are no strict rules used for classification of lncRNAs, except that these sequences have to be longer than 200 bp with open reading frames (ORFs) shorter than 100 amino acids [9]. Identification of long non-coding RNAs is a challenging task, since structurally they are very similar to mRNAs. They are encoded by sequences located in introns of different genes and sometimes even overlapping exons [10]. According to recent studies, as much as 70% of human genome is transcribed, whereas protein coding transcripts cover only about 2% [11]. Some researchers suggest that there are 6736 lncRNA-coding genes in human [12]. Nevertheless, as of March 2015, there were only 127 human lncRNAs that have been functionally annotated, according to the lncRNAdb.org database [13]. Considering this, it seems highly probable that some of those genes may encode currently undiscovered proteins or even play a double role as both the lncRNA-coding and protein-coding entities.

Herein, we aimed to verify a hypothesis that the *CRNDE* gene may encode a protein product, CRNDEP. In order to do that, the gene was investigated *in vitro*, *in situ* and *in silico* on both RNA and protein levels using a variety of molecular, immunohistochemical and computational techniques.

## Results

### Identification of three potential *CRNDE* ORFs followed by the analysis of a secondary structure of hypothetical peptides they encode

**Fig 1:**
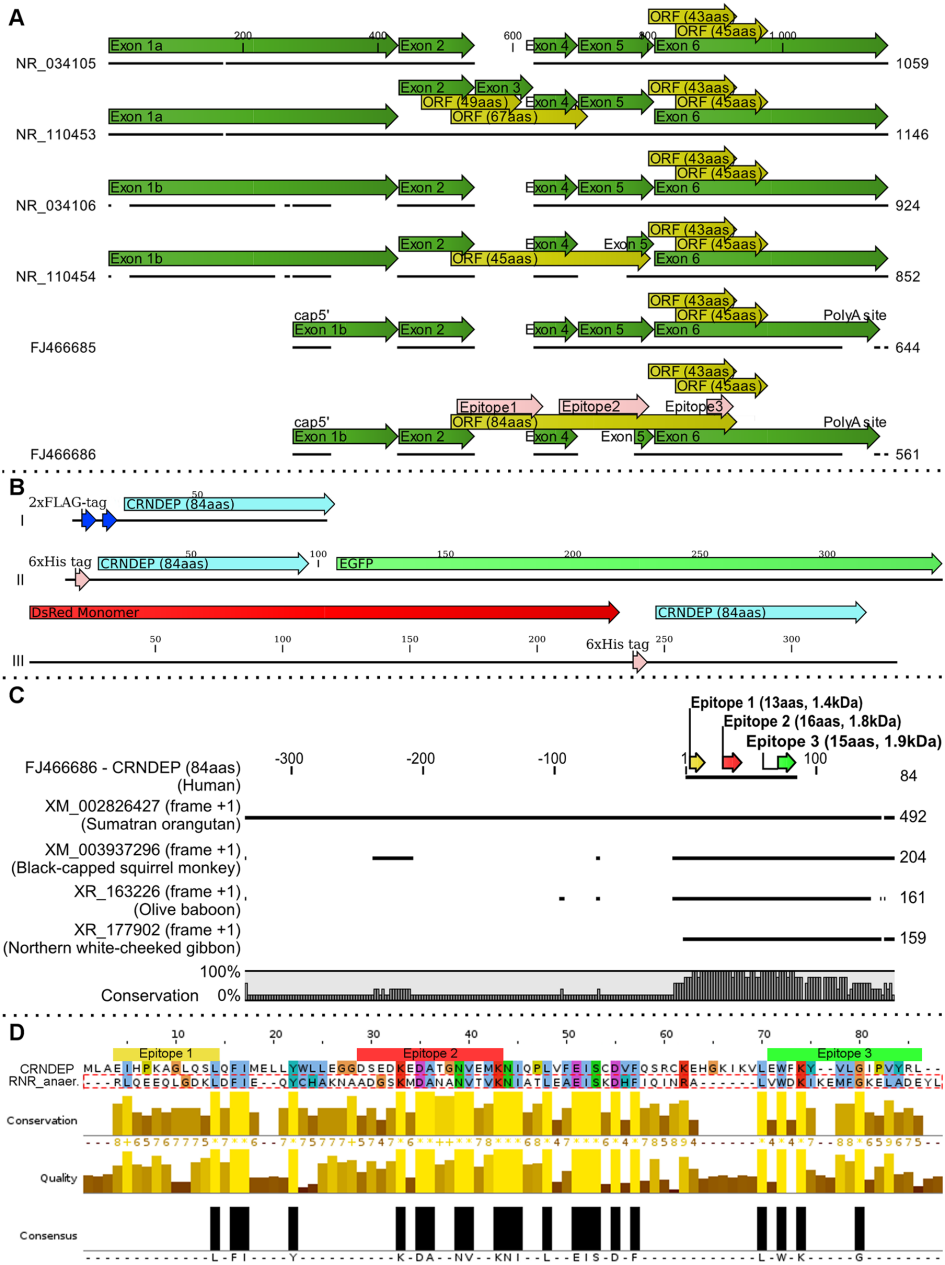
*In silico* studies on *CRNDE* transcripts and the 84aas protein product, CRNDEP. A) A graphical alignment of two complete *CRNDE* transcripts, previously identified by our research team (FJ466686, FJ466685) to four reference RNA sequences (NR_034105, NR_034106, NR_110453, NR_110454) available in GenBank. Our sequences are the most similar to the NR_034106 reference sequence, except for their exons 1b and 6, which are shorter. In addition, exon 5 in the FJ466686 transcript is shortened compared to the other transcripts. This allows for the formation of an alternative 84aas open reading frame (ORF), specific to this particular splice variant. ORFs are shown as yellow arrows, whereas pink arrows represent three epitopes used herein for development of the anti-CRNDEP antibody. B) Structures of three different fusion proteins used in this study after their enforced overexpression in HeLa cells: I—2xFLAG-CRNDEP (120 aas, 14.0 kDa), II—6xHis-CRNDEP-EGFP (346 aas, 39.2 kDa), III—DsRed Monomer-6xHis-CRNDEP (340 aas, 38.5 kDa). C) An alignment of several protein sequences obtained by using the 84aas CRNDEP sequence as a query to translated BLAST (NCBI tblastn). This bioinformatics tool searched the translated nucleotide collection (nr/nt) from all organisms and found hypothetical proteins highly similar to human CRNDEP. Interestingly, all the hits were confined to the Primates order only. D) An alignment between CRNDEP and the N-terminal consensus sequence of RNRs, generated by the Clustal Omega algorithm, showing a significant level of homology and conservation.

Beforehand, we have identified two alternatively spliced *CRNDE* transcripts, previously undescribed (GenBank accession numbers: FJ466685 and FJ466686) [4, 5]. The latter may encode 3 different hypothetical peptides, the longest of which (consisting of 84 amino acids, named CRNDEP herein) is specific to this particular transcript (see Fig 1A). Each of these three hypothetical peptides was analyzed with Globplot2, the algorithm for predicting intrinsic protein disorders, domains and globularity [14], and with a meta server in the BioInfoBank Institute [15]. The results showed that only the longest 84aas peptide was able to form a stable secondary structure with sufficient similarity to other known or hypothetical proteins (see S1 and S3 Figs attached as supporting information).

### Structural and functional analyses performed on the I—TASSER meta server

The sequence of the hypothetical 84aas CRNDEP, submitted to GenBank by our research team (accession number ACJ76642), was used as a query to the I-TASSER meta server [16, 17]. First, the server attempted to predict a secondary structure of the peptide, generating data consistent with our earlier results from the BioInfoBank meta server, especially in respect to alpha helices distribution. Next, solvent accessibility predictions suggested that the epitope characterized by the highest antigenicity is located to the central region of CRNDEP (see S4 Fig).

This analysis was followed by the calculation of five 3-dimensional models. The best model of 84aas CRNDEP had the C-score of -2.35. Typically this parameter ranges from -5 to 2 and the higher value signifies models with higher confidence. The estimated accuracy, measured by TM-score, equaled 0.44±0.14 [16, 17]. TM-score is used for measurement of structural similarity between two proteins. When it is higher than 0.5, a model has a correct topology, while a TM-score lower than 0.17 means a random similarity. Five of ten structural analogs of 84aas CRNDEP, found with the TM-align algorithm, were enzymes (3 oxidoreductases, 1 hydrolase and 1 isomerase). Other 4 analogs were proteins associated with the RhoGTPase pathway and the last one was the GTP binding protein, transducin (see S6 Fig for details).

Prediction of gene ontology (GO) revealed that 84aas CRNDEP may play a role in oxidative stress generation or response, due to its hypothetical oxidoreductase, peroxidase or oxygen-binding activities (GO-scores equaled 0.25, 0.22 and 0.20, respectively). The GO-score is defined as the average weight of the GO-terms, where the weights are assigned based on the global and local similarities between the query and the template protein. Predictions with the GO-score > 0.5 are treated as highly reliable. Considering the high BS-score (1.20), which measures local sequence and structure similarities of hypothetical ligand binding sites (see S7 Fig), it is possible that CRNDEP may have a ligand binding site similar to hemoglobin. On the other hand, the EC-scores (describing the confidence of a predicted Enzymatic Classification (EC) Number) are below the recommended threshold (>1.1), which makes the functional predictions less certain than those concerning ligand binding sites (see S8 Fig for details).

Interestingly, the same bioinformatics tools failed to assign the shorter hypothetical 43aas and 45aas CRNDE peptides to any particular function in the cell.

### Different alignment search tools confirmed the similarity of CRNDEP to other known and hypothetical proteins

The 84aas CRNDE peptide was originally identified by our research team. We also submitted its sequence to GenBank, where it is currently classified under the ID: EAW82818.1 (locus tag: hCG_1815491). By using this sequence as a query to the translated BLAST (NCBI tblastn), we obtained an alignment consisting of human sequences, and also similar predicted proteins in orangutans, gibbons, baboons and squirrel monkeys (see Fig 1C). The 84-amino acid CRNDEP sequence seems to be highly conserved in the Primates order, however, no similar sequences were found in other organisms.

Furthermore, according to the Conserved Domain Database (CDD [18]), a region of CRNDEP encompassing amino acids 14–68 exhibits a statistically significant sequence similarity (E-value = 3.38e-03) to N-terminal regions of ribonucleoside-triphosphate reductases (RNR), bacterial enzymes operating under anaerobic conditions [19]. Driven by this fact, we aligned CRNDEP and 77 amino acids of the N-terminal consensus sequence of RNRs using the Clustal Omega algorithm. The outcome confirmed a significant level of homology and conservation between these two proteins, especially in the central part of CRNDEP (see Fig 1D).

### Antigenicity predictions for CRNDEP with three different algorithms

Additional *in silico* research, with three different methods for peptide antigenicity prediction, was carried out to identify the most antigenic regions of CRNDEP. The results obtained with two methods [20, 21] were very similar to each other, and suggested that the best epitope is located between the 25th and 49th amino acid residue (see S5 Fig). This conclusion was consistent with the solvent accessibility predictions shown in S4 Fig According to the COBEpro (Continuous B-cell Epitopes) method [21], predicted probability of antigenicity equaled 0.112. On the contrary, the outcome of the third method [22], utilized by the EMBOSS Antigenic algorithm, was significantly different. Although the accordance between the antigenicity prediction methods was not full, a similar outcome of two methods may suggest their correctness.

### A synthesis of custom-made polyclonal anti-CRNDEP antibodies in rabbits

**Fig 2:**
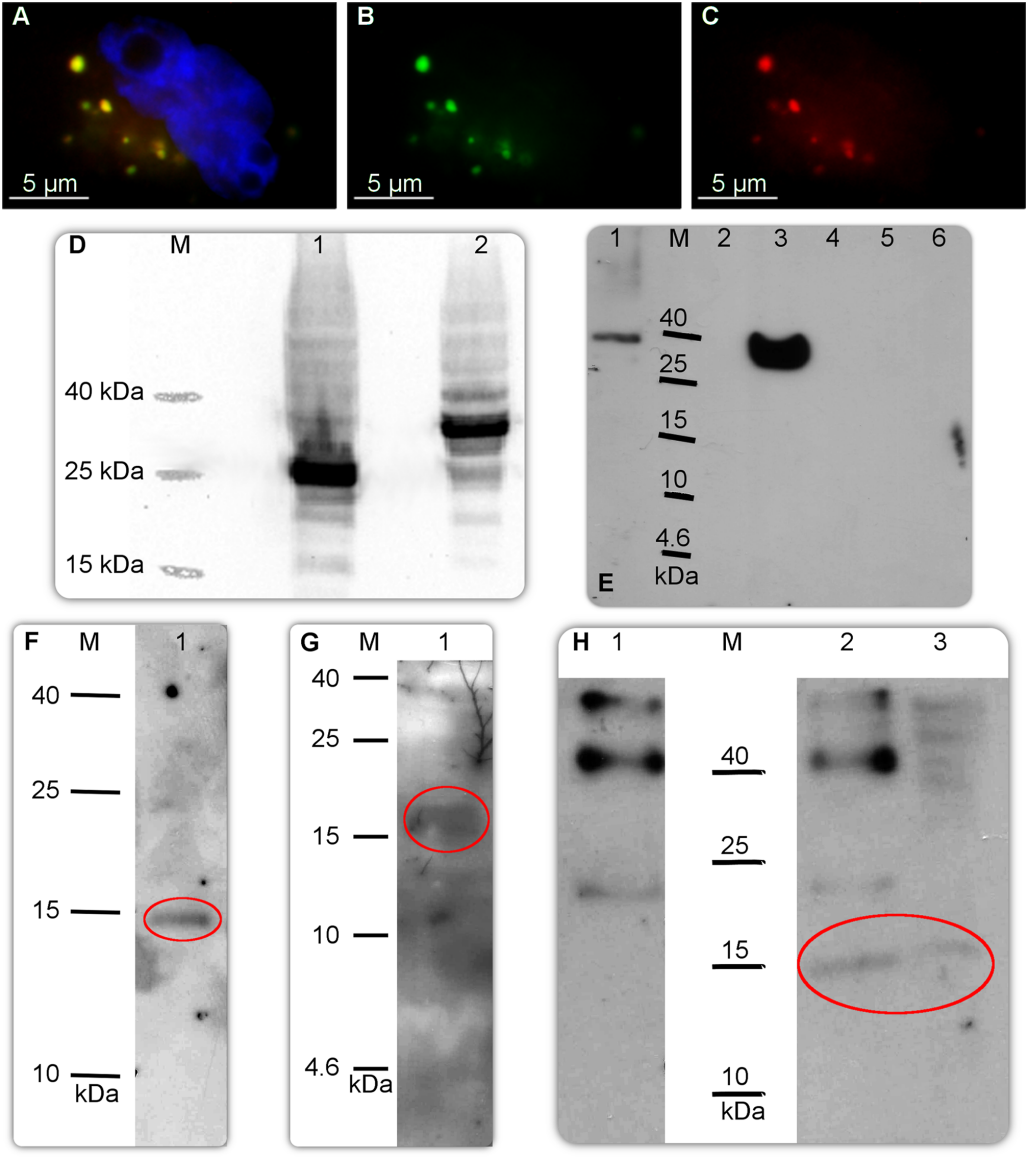
Molecular studies on CRNDEP. A) Simultaneous overexpression of the 6xHis-CRNDEP-EGFP and DsRed Monomer-6xHis-CRNDEP fusion proteins in HeLa cells, visualized under a fluorescence microscope. The former protein glows green and the latter glows red in these conditions. Yellow glow is caused by a co-localization of these two fusion proteins. Nuclei were stained blue with DAPI. The same shot with only the green (B) or the red (C) channel shown. D) Western blot-based verification of the size of the 6xHis-CRNDEP-EGFP fusion protein. M—Spectra Multicolor Low Range Protein Ladder (Thermo-Fisher Scientific), 1—the EGFP reporter protein (26.9 kDa), 2—6xHis-CRNDEP-EGFP (39.2 kDa). E) Western blot-based verification of the specificity of our custom-made polyclonal anti-CRNDEP antibody. M—Spectra Multicolor Low Range Protein Ladder; 1—DsRed Monomer-6xHis-CRNDEP (340 aas, 38.5 kDa); 2—purified 14 kDa protein containing the 6xHis tag, 1.4 μg (a negative control of the antibody's specificity, non-commercial); 3—6xHis-CRNDEP-EGFP (346 aas, 39.2 kDa); 4—empty; 5—EGFP (239 aas, 26.9 kDa, a negative control); 6—DsRed Monomer (232 aas, 26.2 kDa, a negative control). A loading control (the PVDF membrane used in this experiment, stained with Ponceau S) is shown in S13 Fig. F–G) Detection of the overexpressed 2xFLAG-CRNDEP protein (~14 kDa) in a total protein lysate from 0.25 million HeLa cells with either the anti-FLAG (F) or anti-CRNDEP (G) antibody. H) Immunoprecipitation of 2xFLAG-CRNDEP using the anti-CRNDEP antibody (2) and control IgG (1) (both from a rabbit). A total protein lysate before immunoprecipitation was loaded for comparison (3). After precipitation, the 2xFLAG-CRNDEP protein was detected on the PVDF membrane using the anti-FLAG antibody. The correct bands in Fig F–H are encircled.

A custom-made rabbit primary polyclonal anti-CRNDEP antibody was ordered in Abgent, Inc. (San Diego, CA, USA). The company used three different epitopes (see Table 1 for sequences). The Epitopes 1 and 3 were located to the N and C terminus of the peptide, respectively. The Epitope 2 lay in the central region of CRNDEP (see Fig 1D and S2 Fig), and it seemed to be the best antigen available (see S5 Fig). It should be stressed that the Epitope 2 is encoded by the mRNA region spanning the junction between exons 4 and 5 (see Fig 1A). Thus, this epitope is specific only to the 84aas protein product of *CRNDE*. The custom antibody development was carried out in two rabbits per each of the three epitopes. Specificity of obtained antibodies was successfully verified only for the Epitope 2 in Western blot studies utilizing the evaluated primary antibody and the HRP-conjugated donkey anti-rabbit secondary polyclonal antibody (see Fig 2E). The results of ELISA tests performed by Abgent, Inc. were also correct, confirming a strong and specific immunological reaction of the rabbit against the Epitope 2. In those tests, the optical density obtained for the immunized serum was higher by 1.89 O.D. units compared to the same serum before immunization (both sera were diluted 4000 times).

**Table 1:** Sequences of primers used for PCR, sequencing and Real-Time PCR, followed by sequences of a TaqMan probe, shRNAs, and CRNDEP epitopes used in the present study.

Primer name	Sequence [5' → 3']^1^ or [NH_2_ → COOH]
L388-SacI PCR primer	CCTGAGCTCCTATGAGAGGATCGCATCAC
L388-SmaIv2 PCR primer	GGTCCCGGGCAATTAAGCTTTAGTCTATAAAC
L388BglII PCR primer	GGTAGATCTCTATGAGAGGATCGCATCAC
L388SmaI PCR primer	GGTCCCGGGCATTAAGCTTTAGTCTATAAAC
RedL388F PCR primer	AACCACAACGAGGACTAC
RedL388R PCR primer	GGACAAACCACAACTAGAATG
L388wewF forward sequencing primer	CTGTACTGGCTATTGGAAGG
L388wewR reverse sequencing primer	TAAACCACTCGAGCACTTTG
U6prom-F sequencing primer	GACTATCATATGCTTACCGT
SV40rev sequencing primer	CATACTTCTGCCTGCTGGG
LOCrtF—Real-Time PCR forward primer (universal)	AATTCATCCCAAGGCTGGTC
LOCrt35R —Real-Time PCR reverse primer (specific to the CRNDEP-coding transcript, FJ466686)	TTCCAGTGGCATCCTCCTTA
LOCrt4wR—Real-Time PCR reverse primer (specific to non-CRNDEP-coding transcripts, e.g., FJ466685)	GCACTCACAATGAGTCATCTG
*CRNDE*-specific TaqMan probe (universal)	CCTTCCAATAGCCAGTACAGTAGCTCC
SH1 sense strand^2^	GATCGGGAGCTACTGTACTGGCTATTGGAAGGAGTCAAGAGCTCCTTCCAATAGCCAGTACAGTAGCTCCTTTTTTGA
SH1 complementary strand^2^	AGCTTCAAAAAAGGAGCTACTGTACTGGCTATTGGAAGGAGCTCTTGACTCCTTCCAATAGCCAGTACAGTAGCTCCC
SH2 sense strand^2^	GATCGAGAAGAAGGTTAAGCTGTATTTGATTGCCTCAAGAGGGCAATCAAATACAGCTTAACCTTCTTCTTTTTTTGA
SH2 complementary strand^2^	AGCTTCAAAAAAAGAAGAAGGTTAAGCTGTATTTGATTGCCCTCTTGAGGCAATCAAATACAGCTTAACCTTCTTCTC
SH3 sense strand^2^	GATCGGAAGATAAGGAGGATGCCACTGGAAATGTTCAAGAGACATTTCCAGTGGCATCCTCCTTATCTTCTTTTTTGA
SH3 complementary strand^2^	AGCTTCAAAAAAGAAGATAAGGAGGATGCCACTGGAAATGTCTCTTGAACATTTCCAGTGGCATCCTCCTTATCTTCC
SH SCR (scrambled, negative control) sense strand^2^	GATCGGGAGCAATATCGTGGATGAAACGGTGAAATCAAGAGTTTCACCGTTTCATCCACGATATTGCTCCTTTTTTGA
SH SCR (scrambled, negative control) complementary strand^2^	AGCTTCAAAAAAGGAGCAATATCGTGGATGAAACGGTGAAACTCTTGATTTCACCGTTTCATCCACGATATTGCTCCC
CRNDEP Epitope 1	EIHPKAGLQSLQ
CRNDEP Epitope 2	DSEDKEDATGNVEMK
CRNDEP Epitope 3	EWFKYVLGIPVYRL

^1^) Restriction enzyme recognition sites inserted in primer overhangs were underlined.

^2^) shRNA-coding sequences: sense regions were marked with a wavy line, whereas antisense regions were indicated with a double wavy line; sticky ends, specific to BamHI/HindIII digestion, were double-underlined.

### Studies on the localization of overexpressed CRNDEP in HeLa cells

Identification of a cellular localization of CRNDEP started with a control study, in which two fusion proteins, 6xHis-CRNDEP-EGFP and DsRed Monomer-6xHis-CRNDEP (see Fig 1B) were overexpressed in HeLa cells simultaneously, resulting in a full co-localization in some kind of granules outside the nucleus (see Fig 2A–2C). Thus, evidently, the observed localization of the fusion proteins depended on CRNDEP, and not on the reporter protein.

In the next step, total protein lysates from HeLa cells, transfected with either the empty pEGFP-N1 vector or the pEGFP-N1_CRNDEP plasmid, were analyzed by tricine-SDS-PAGE and Western blotting with the use of the goat primary anti-GFP antibody and rabbit secondary anti-goat HRP-conjugated antibody. The results proved that the size of both EGFP and His-CRNDEP-EGFP proteins was correct and consistent with earlier predictions *in silico* (see Fig 2D).

**Fig 3:**
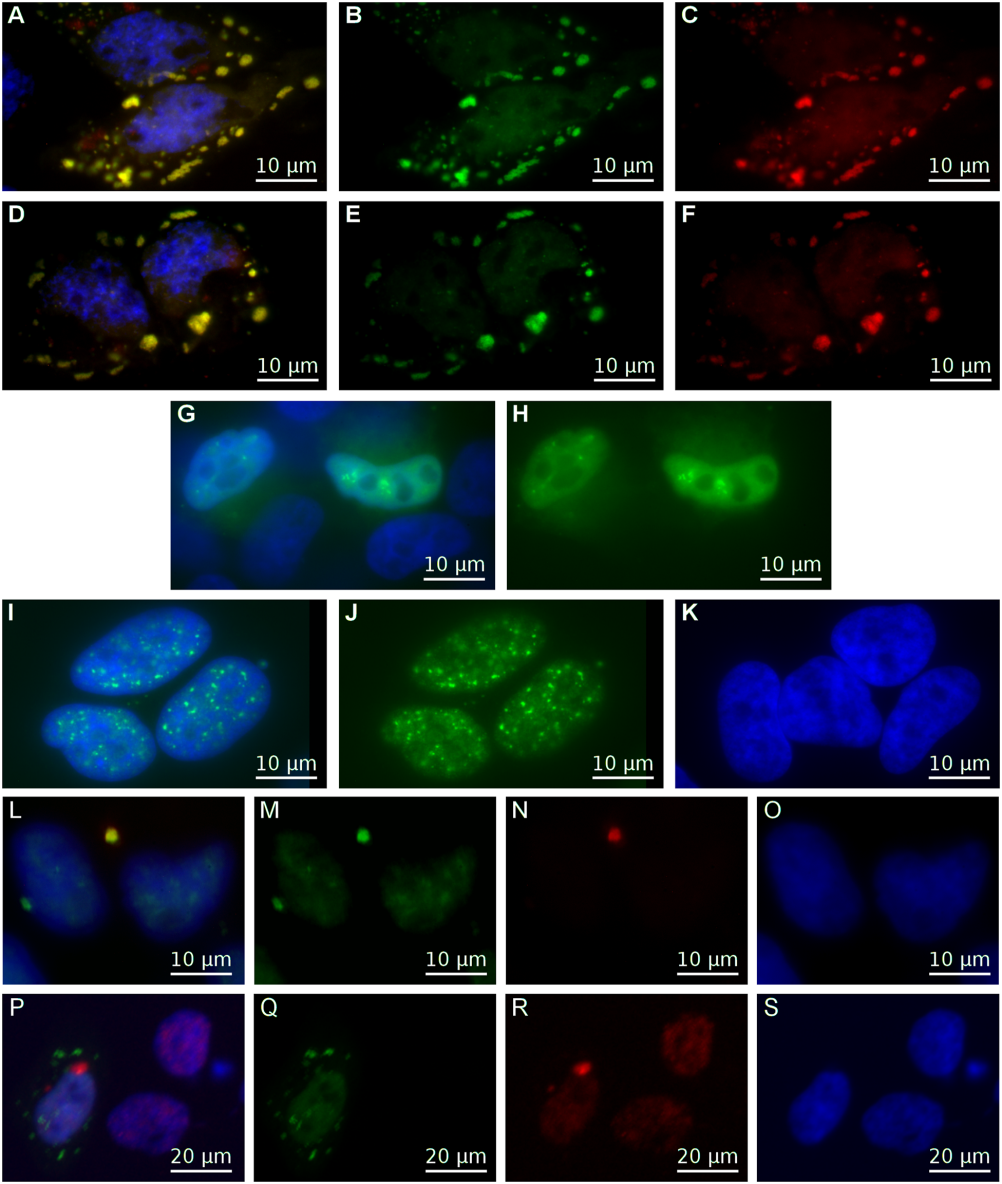
Cellular localization experiments. A–H) Stress granules—localization studies of CRNDEP in a fusion with a fluorescent tag. The EYFP-TIA-1 fusion protein (specific to stress granules) glowed green, the DsRed Monomer-6xHis-CRNDEP fusion protein glowed red and nuclei were stained blue with DAPI. HeLa cells after co-transfection with both the pDsRed Monomer-C1_CRNDEP and pEYFP-TIA-1 plasmids (not treated with sodium arsenite) (A); the same shot with only the green (B) or red (C) channel shown. When this co-transfection was followed by the treatment with sodium arsenite (D), stress granules appeared as well; the same shot with only the green (E) or red (F) channel shown. On the contrary, a transfection of HeLa cells with the pEYFP-TIA-1 plasmid only (G) did not cause the formation of stress granules; the same shot with only the green channel shown (H). I–K) The results of immunofluorescence studies in HeLa cells. Endogenous CRNDEP was immunostained green using the custom-made primary rabbit anti-CRNDEP antibody in combination with the secondary fluorescein (FITC)-conjugated anti-rabbit antibody (I); the same shot with only the green channel shown (J). In order to check the reaction specificity, the cells were incubated with the secondary antibody only. The lack of a green glow is a proof that the staining is specific (K). L–O) HeLa cells after overexpression of the fusion protein DsRed Monomer-CRNDEP, not treated with sodium arsenite. This fusion protein glowed red under a fluorescence microscope, localized to stress granules and was absent in the nucleus (N). By contrast, endogenous CRNDEP (stained green with FITC) was present predominantly in the nucleus, where it formed grains (M). Overlapping of green and red signals in stress granules (L) proved that the anti-CRNDEP antibody is capable of specifically detecting both the endogenous and the artificially overexpressed variants of CRNDEP. P–S) HeLa cells transfected with the pYFP-TIA-1 plasmid and also treated with sodium arsenite. Stress granules were visible as green dots outside the nucleus, which was stained blue with DAPI. No co-localization between endogenous CRNDEP (red) and TIA-1 (green) was observed (P); the same shot with only the green (Q), red (R) and blue (S) channel shown.

The two CRNDEP-containing fusion proteins were then examined for their localization within five cellular compartments, by either specific staining or co-expression with appropriate markers (shown in brackets): Golgi apparatus (pDsRed-Monomer-Golgi plasmid (Clontech, Mountain View, CA, USA)), mitochondria (MitoTracker Red CMXRos dye (Life Technologies, Carlsbad, CA, USA), peroxisomes (SelectFX Alexa Fluor 488 Peroxisome Labeling Kit (Life Technologies)), processing bodies (P-bodies) (mRFP-Dcp1a plasmid (non-commercial)) and stress granules (pEYFP-TIA-1 plasmid (non-commercial)). Co-localization occurred in the stress granules only (see Fig 3A–3F and S9 Fig). Moreover, overexpression of CRNDEP caused the formation of these granules, no matter if the cells were treated with sodium arsenite, an oxidative stress-inducing agent (Fig 3D) or not (Fig 3A). In contrast, a transfection of HeLa cells with the pEYFP-TIA-1 plasmid only did not lead to the formation of stress granules (Fig 3G and 3H).

### Proving the presence of CRNDEP in a variety of human tissues

**Fig 4:**
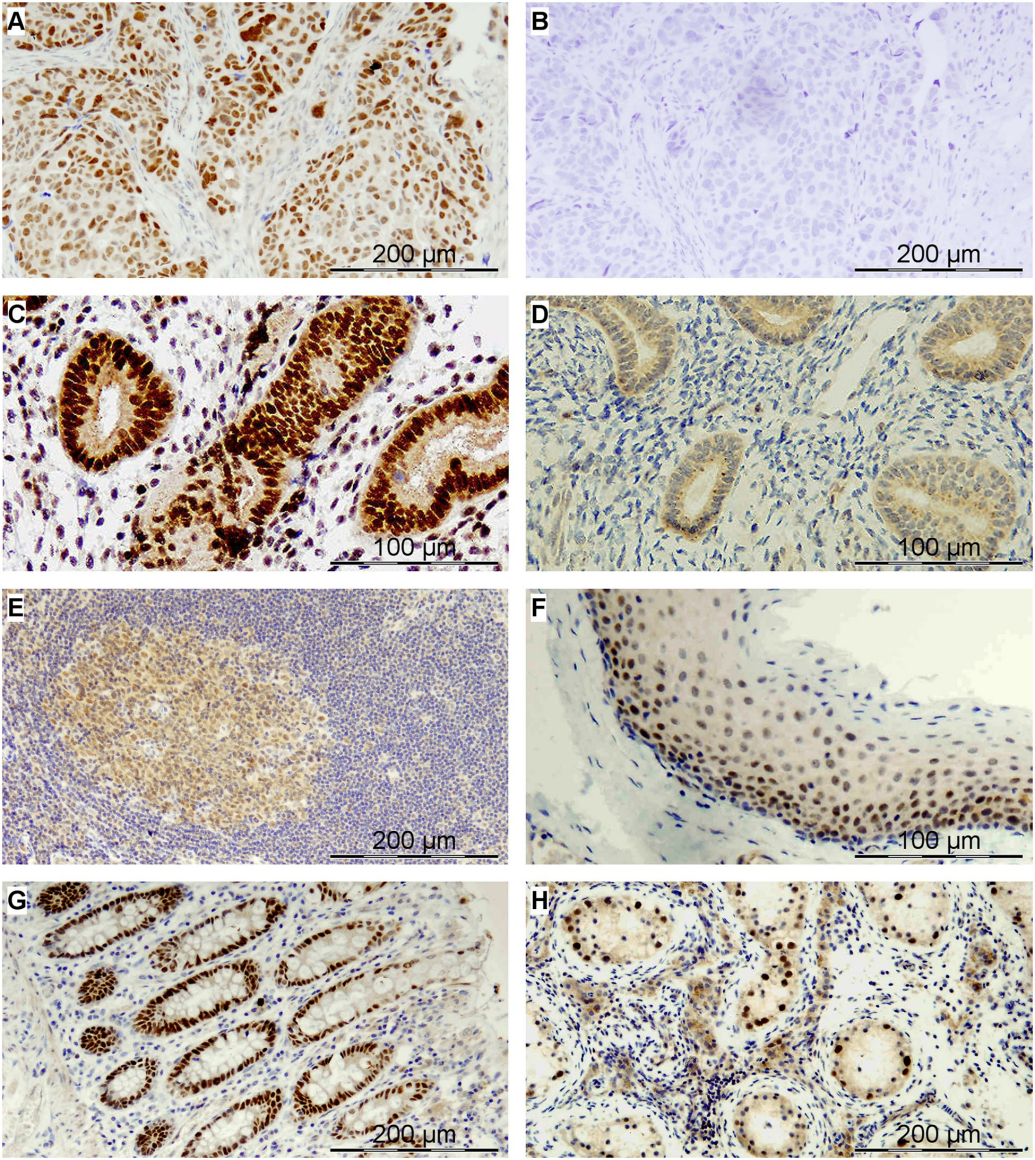
The endogenous CRNDEP peptide expression in different human tissues evaluated by immunohistochemical stainings. A) Epithelial ovarian cancer (serous carcinoma) with heterogeneous nuclear expression; B) The same section of ovarian carcinoma incubated with a blocking peptide; C) Normal proliferative phase endometrium with strong nuclear expression within the glandular epithelium and heterogeneous staining in stromal cells; D) Atrophic endometrium with negative staining in the nuclei; Normal tonsil (E-F) with heterogeneous (weak to moderate) nuclear expression in the germinal center (E) and strong nuclear expression in the parabasal layer of the squamous epithelium (F); G) Normal intestine: strong nuclear expression in intestinal crypts; H) Seminiferous tubules of an atrophic human testis: strong nuclear expression in spermatocytes.

The development of anti-CRNDEP antibody allowed for evaluating the cellular localization of endogenous CRNDEP in a variety of normal and malignant human tissues with the use of immunohistochemical (IHC) methods (see Fig 4). The set of samples comprised FFPE sections of the ovarian carcinoma and normal endometrium, tonsil, intestine and testis. Frozen sections of the ovarian carcinoma were also evaluated (see S10 Fig). Unlike the fusion proteins consisting of CRNDEP and a fluorescent marker, endogenous CRNDEP was characterized by strong nuclear expression in tissues with a high turnover rate, like ovarian cancer cells, intestinal crypts, parabasal layer of the squamous epithelium, spermatocytes or proliferative phase endometrium. Upon addition of the blocking peptide, no immunostaining was observed in either FFPE or frozen tissue sections (see Fig 4B and S10B Fig). The nuclear localization of endogenous CRNDEP was also confirmed in immunofluorescence studies (see Fig 3I and 3J).

### Evaluating the presence of endogenous CRNDEP in stress granules

In order to explain the apparent discrepancy related to a cellular localization of endogenous and artificially overexpressed CRNDEP, we conducted two additional experiments under a fluorescence microscope.

Firstly, HeLa cells were transfected with the pEYFP-TIA-1 plasmid to visualize the stress granules. Then, the cells were treated with sodium arsenite to induce oxidative stress followed by immunofluorescence staining with the use of our anti-CRNDEP antibody. This experiment showed that the stress granules did not contain endogenous CRNDEP (see Fig 3P–3S).

Next, we transfected HeLa cells with the plasmid encoding the fusion protein DsRed Monomer-6xHis-CRNDEP, and then performed an immunofluorescence-based detection of both overexpressed and endogenous forms of CRNDEP using the anti-CRNDEP antibody. The results were in step with our earlier observations, since endogenous CRNDEP was the only form detectable inside the nucleus (see the presence of green grains and the lack of red ones in Fig 3M and 3N). At the same time, enforced overexpression of DsRed Monomer-6xHis-CRNDEP caused the formation of stress granules with concomitant accumulation of this fusion protein within these structures. A yellow glow inside the stress granules (see Fig 3L) proved the specificity of the anti-CRNDEP antibody, which was able to detect both the endogenous (nuclear) and overexpressed (cytoplasmic) forms of CRNDEP.

### Additional verification of specificity of the anti-CRNDEP antibody

Considering that the anti-CRNDEP antibody was custom-made, we had to check its specificity in several ways, including the localization (Fig 3L–3O) and IHC (Fig 4B and S10B Fig) experiments. Besides, we also succeeded in detecting overexpressed CRNDEP fused to either EGFP or DsRed Monomer by Western blotting (see Fig 2E). After improving the sensitivity of the Western blot method, as suggested by Suzuki et al. [23], we were able to detect the overexpressed 2xFLAG-CRNDEP fusion protein (~14 kDa) in a total protein lysate from HeLa cells with either the commercial anti-FLAG or our anti-CRNDEP antibody (see Fig 2F and 2G). Furthermore, we managed to carry out successful immunoprecipitation of the 2xFLAG-CRNDEP protein using the anti-CRNDEP antibody immobilized on a resin, followed by immunoblotting with the FLAG-specific antibody (see Fig 2H). Nevertheless, endogenous CRNDEP still remained undetectable on Western blots.

**Fig 5:**
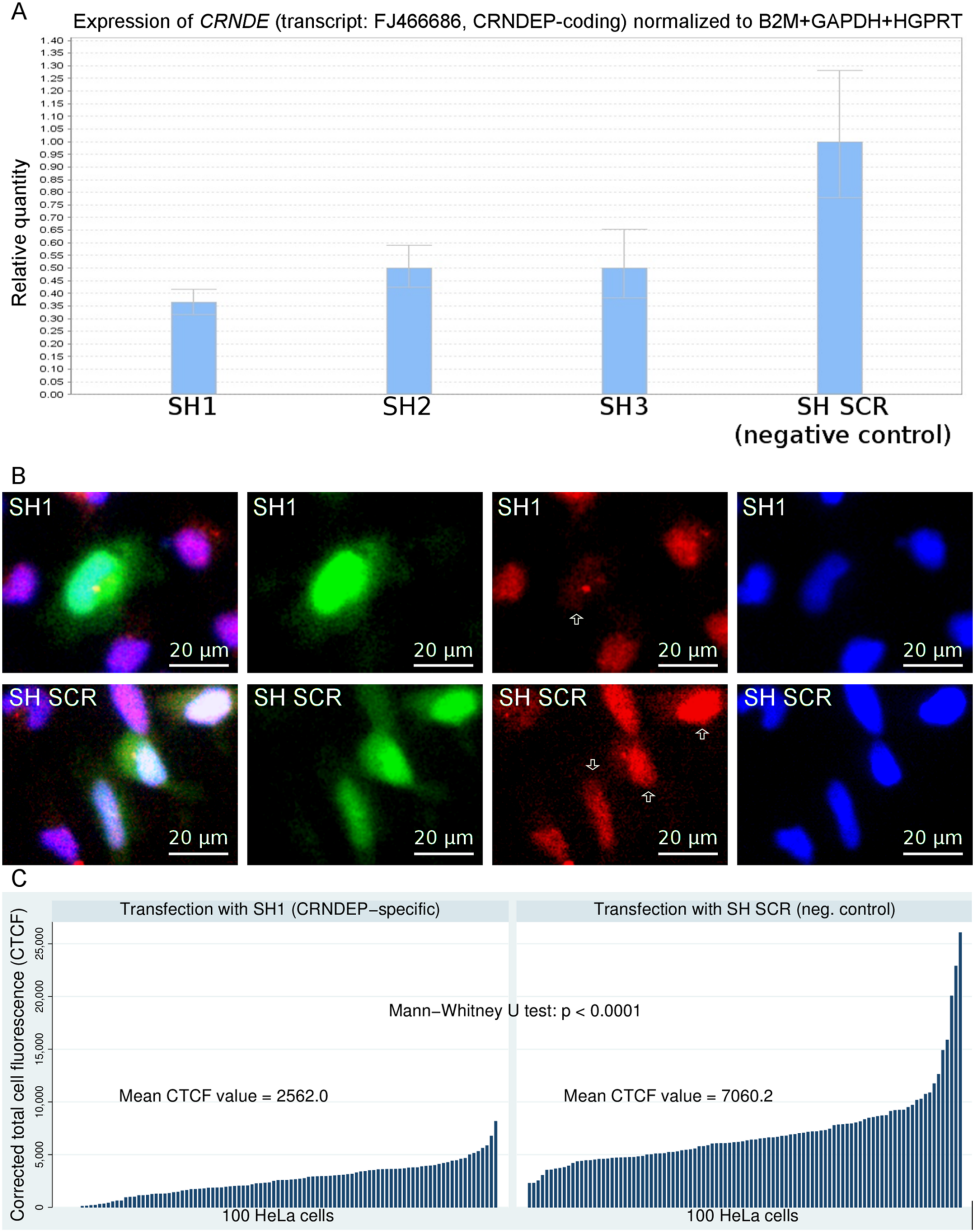
Evaluation of shRNA-mediated knockdown of *CRNDE*. The effects of *CRNDE* gene silencing were evaluated at either mRNA (A) or protein level (B-C). The strongest decrease in the amount of the CRNDEP-coding transcript (by ~65%) was observed for the SH1 silencing construct (A). The effects of this knockdown were detectable at the protein level as well (B, C), leading to a statistically significant decline in the amount of CRNDEP (red signal) in the cells transfected with the silencing construct (green signal). As expected, such a correlation did not occur in the cells transfected with the construct encoding a control (scrambled) shRNA molecule (SH SCR). The transfected cells are marked with white arrows.

Given the problems with detecting endogenous CRNDEP on Western blots, we decided to check whether the siRNA-mediated decrease in mRNA level of the corresponding *CRNDE* transcript affects the level of CRNDEP in HeLa cells. Each of the three silencing constructs prepared for this purpose was able to knockdown the *CRNDE* gene expression by at least 50% (see Fig 5A), when measured by Real-Time qPCR 48 hours after a transient transfection. The cells transfected with the most efficient *CRNDE*-silencing shRNA (SH1) and the control plasmid (SH SCR) were subjected to an immunofluorescence evaluation with the use of our anti-CRNDEP antibody. The statistical analysis of the corrected total cell fluorescence (CTCF) values in a red channel (corresponding to the *CRNDE* expression) showed a ~ 60% decrease of the CRNDEP level in the knocked-down cells compared to the control ones. The difference in *CRNDE* expression was statistically significant with a p-value < 0.0001 (see Fig 5B and 5C), thus indirectly proving that CRNDEP is indeed encoded by the *CRNDE* gene.

## Discussion

Herein, we have demonstrated that the *CRNDE* gene may encode a nuclear peptide, CRNDEP, which is likely involved in the cell proliferation, since its endogenous expression is elevated in highly proliferating tissues. Additionally, CRNDEP may also stimulate the formation of stress granules when artificially overexpressed.

### *CRNDE* may possibly encode both lncRNAs and protein products

It seems likely that *CRNDE* transcripts may play divergent roles in the cell as both protein-coding and lncRNA-coding entities. A similar mechanism has already been described for other genes, e.g., the Steroid Receptor RNA Activator 1 (*SRA1*), which encodes lncRNA SRA. Additionally, some *SRA1* transcripts may encode a protein product SRAP [24]. The balance between protein-coding and non-coding transcripts of *SRA1* is regulated by alternative splicing [25], and any misregulation promotes cancer development [26]. It is conceivable that the *CRNDE* gene may be regulated in a similar way, given that this gene encodes over 10 different splice variants, according to the AceView database [27]. Moreover, some of the transcripts, that have retained intronic sequences are highly enriched in the nucleus, which is a hallmark of lncRNAs. On the other hand, the splice variants that contain only exons are mainly detectable in the cytoplasm, like protein-coding transcripts [6]. It is also possible that, like in case of *SRA1*, the imbalance between different *CRNDE* transcripts may be associated with carcinogenesis. Accordingly, in two studies by Furney et al. and Maguire et al., mutations in the *SF3B1* gene, encoding a component of the spliceosome, were shown to be connected to differential alternative splicing of *CRNDE* pre-RNA in uveal melanomas [28] and breast cancers [29].

### The amino acid sequence of CRNDEP seems to be highly conserved in the Primates order

By using the tblastn algorithm we discovered that the 84-amino acid sequence of CRNDEP is highly conserved among Primates and absent in other species. This *in silico* result may suggest that CRNDEP is exclusively expressed in higher mammals. Such a hypothesis seems to be in line with the observations by Lin et al. [30] and Ellis et al. [6], showing that the *CRNDE* gene is expressed in both fetal and mature brains, where it exhibits highly specific spatial expression within different substructures. Considering that Primates have the most sophisticated brains from among living creatures, the existence of transcripts/proteins expressed exclusively in this order becomes justifiable. It is worth noting that the hypothetical animal CRNDEP orthologs seem to be longer than human CRNDEP, however, the corresponding region of 84 amino acids is highly conserved in all the species (the query sequence coverage ranged from 92–94%, while the pairwise sequence identity equaled 86–94%). Interestingly, the sequence of 15aas oligopeptide (named the Epitope 2 herein), located within that conserved region and used for development of our anti-CRNDEP antibody, did not change between orangutans, gibbons, baboons and humans during 25 million years of independent evolution [31].

### Evaluation of the cellular localization of CRNDEP

The endogenous CRNDEP peptide exhibits strong nuclear expression in cancer cells and normal tissues (or their components) characterized by a high proliferation rate. On the other hand, our localization experiments in HeLa cells suggested that CRNDEP locates to the stress granules and stimulates their formation, similarly to sodium arsenite, yet independently. Nevertheless, it needs to be stressed that in our localization studies CRNDEP was highly overexpressed and fused to either GFP or DsRed Monomer, two relatively big fluorescent markers. It is likely that strong, enforced overexpression of CRNDEP interferes with the proper function of this peptide, which eventually results in its non-physiological cellular localization and the formation of stress granules. The underlying mechanism has to be elucidated in future studies. An analogous difference between the localization of the endogenous protein and its artificially overexpressed fusion with GFP has already been described by Abe et al. for the Rho1p GTPase in yeast [32]. Similarly to CRNDEP, Rho1p is smaller (209 aas, 23.2 kDa) than GFP, thus supporting the hypothesis that a big fluorescent tag may interfere with a proper function of the studied protein, especially if it is relatively small.

### Confirmation of the specificity of the anti-CRNDEP antibody

The scientific usefulness and specificity of our custom-made anti-CRNDEP antibody was successfully verified in a few different ways. In IHC experiments, this antibody was able to detect endogenous CRNDEP in various frozen and FFPE tissue sections, thus not only confirming its analytical value, but also giving the experimental evidence of the CRNDEP existence in human cells. By using the Epitope 2 as a blocking peptide during immunohistochemical stainings, we showed that the antibody is completely inactivated by this epitope, which proved the specificity of antigen-antibody interactions. Our cellular localization experiments also demonstrated that the anti-CRNDEP antibody is capable of specifically detecting both endogenous and artificially overexpressed forms of CRNDEP. In addition, we managed to observe a statistically significant decrease in the amount of CRNDEP in HeLa cells after a transient transfection with our most efficient CRNDE-silencing construct SH1. This result was the proof that there is a correlation between the amount of CRNDEP and the expression level of the relevant transcript, as well as the ultimate confirmation of specificity of the anti-CRNDEP antibody developed by our research group. Nonetheless, it needs to be stressed that a potential cross-reaction of the CRNDEP-specific antibody with another protein containing a similar epitope cannot be entirely excluded, since we do not know yet the entire human proteome.

Remarkably, the expression of CRNDEP in the testis does not seem to be significantly higher compared to the colon, as it was shown in Fig 4. This result may seem contrary to the data from gene expression microarray, presented by Ellis et al. [6], which indicated that the expression of *CRNDE* is ~300 times higher in spermatozoa than in the colon epithelium. We managed to explain this apparent discrepancy by performing Real-Time qPCR in various normal human tissues, using our personally-designed TaqMan assay, specific to the CRNDEP-coding transcript only (see S12 Fig). As expected, the *CRNDE* expression in the testis (15) is, indeed, one of the highest. Nevertheless, the ratio between the expression in the testis and the colon (21) clearly depends on a reference gene used for normalization, ranging from several times (*HGPRT*) to several hundred times (*B2M*). This result shows that a method of normalization strongly affects the final outcome, because the expression of different housekeeping genes unambiguously differs between tissues. It is also important to note that the colon is composed, to much larger extent than the testis, of different tissues, including the smooth muscle cells, the connective tissue, and the abundant epithelial tissue, characterized by a low proliferation rate. In these tissues, the expression of CRNDEP may be significantly lower than in the intestinal crypts. *In situ* studies, such as immunohistochemical ones, provide detailed information on the antigen localization in contrast to other methods. Last but not least, our IHC specimen came from an atrophic testis, in which the expression of CRNDEP may potentially be lower than in the normal testis.

As to the Western blot studies, we initially encountered some difficulties in detecting small CRNDEP fusion peptides, even though these peptides were highly overexpressed. Finally, we have been able to partially improve the results of immunoblotting by introducing some crucial modifications to this technique, as suggested in Ref. [23], i.e., a selection of a membrane with a non-standard size of pores, optimization of the transfer time, as well as an altered protein fixation protocol (see the Methods section). All these changes enabled us to successfully detect the overexpressed 2xFLAG-CRNDEP fusion protein with either the anti-FLAG or anti-CRNDEP antibody. On both membranes single bands of a similar size were detected, thus confirming the specificity of anti-CRNDEP antibody. Another supporting evidence came from our immunoprecipitation studies, which utilized both of the aforementioned antibodies and also gave positive results. It is noteworthy that we still fail to visualize endogenous CRNDEP on Western blots. Considering that the level of CRNDEP-coding transcript in the control cells is several thousand times lower than in the cells after enforced overexpression (see S11 Fig), it seems highly probable that some further improvements in terms of sensitivity are needed to detect endogenous CRNDEP on Western blots.

Interestingly, some recent studies suggest that the initiation of translation is one of the critical steps during the induction of gene silencing by RNA interference [33, 34]. Given the unequivocal decrease of *CRNDE* expression at mRNA level after its shRNA-mediated knockdown, these reports indirectly support our thesis that *CRNDE* is a protein-coding gene.

### The *CRNDE* gene—speculations on the function of its protein product

The expression of *CRNDE* was shown to be highly diverse between different tissues. It also depends on the developmental stage of the organism, being the highest in early human ontogenesis [6]. The authors showed as well that *CRNDE* displays highly specific spatial expression within the substructures of the mature human, rat, and mouse brain. In step with these findings, Trimarchi et al. [35] reported that the *CRNDE* gene is involved in development of the retinal ganglion in mice, whereas Lin et al. [23, 30] suggested its role in neuronal lineage differentiation. Besides, *CRNDE* is supposed to be required to maintain the pluripotency of embryonic stem cells, and thus it is potentially associated with cancer development [7].

A few research groups reported that *CRNDE* is upregulated in colorectal cancers [2, 36, 37] and some blood and brain malignancies [6]. In accordance to these findings, we also identified *CRNDE* as a novel molecular marker of poor prognosis in ovarian cancer [4, 5]. Recently, Ellis et al. [38] demonstrated the existence of a few, evolutionarily conserved, nuclear *CRNDE* transcripts, like gVC-In4, which have the ability to retain intronic sequences. They also found that the knockdown of gVC-In4 in HCT116 cells resulted in deregulation of genes associated with insulin-mediated signaling in a pattern suggestive of the decreased Warburg effect [38, 39]. In other words, *CRNDE* expression may potentially promote molecular changes responsible for the switch to aerobic glycolysis, being one of cancer hallmarks.

The exact function of CRNDEP remains to be discovered. The IHC results presented herein suggest that this peptide may be involved in the cell proliferation, because its expression is significantly elevated in highly proliferating tissues. This observation is consistent with the hypothesis by Ellis et al. [6] that *CRNDE* may be a target of the MYC regulatory pathway, being associated with the cell cycle progression and malignant transformation [40]. Interestingly, high CRNDEP expression observed in the testis supports another conception by Ellis et al. [6] that *CRNDE* may contribute to gametogenesis. On the other hand, our *in silico* calculations carried out on the I—TASSER meta server have classified this peptide as a hypothetical enzyme with potential oxidoreductase/peroxidase activity, conceivably involved in oxygen metabolism. Despite a very preliminary character of these analyses and the lack of supporting data obtained *in vitro*, a quite good quality of the 3D model of CRNDEP (TM-score = 0.44±0.14) suggests that they may be biologically relevant to some extent. This hypothesis is even more convincing in light of our cellular localization studies showing the relationship between the enforced CRNDEP overexpression and the formation of stress granules in HeLa cells.

Interestingly, the region spanning residues 11–68 of the 84aas CRNDE peptide, is similar to the ribonucleoside-triphosphate reductase (RNR) from *Haemophilus parainfluenzae* (the query sequence coverage and pairwise sequence identity equal 82% and 35%, respectively). This bacterial protein belongs to the family of glycine-radical-containing oxidoreductases, being oxygen-sensitive and operating under anaerobic conditions [18, 19]. A good quality alignment obtained for these two proteins may support the predicted CRNDEP structure. Nevertheless, the activity of the bacterial enzyme cannot be directly linked to the structural motif of human CRNDEP, because the latter lacks the Gly-radical site, being essential for activation of RNRs [41].

## Conclusions

In summary, we have shown here that the *CRNDE* gene encodes CRNDEP, the nuclear peptide possibly involved in the cell turnover, since its endogenous expression is elevated in highly proliferating tissues. When artificially overexpressed, CRNDEP seems to stimulate the formation of stress granules and localize to them. In the meantime, another research team characterized *CRNDE* as the lncRNA-coding gene [1, 2]. Given our and their results suggesting its role in, e.g., carcinogenesis, developmental biology, and differentiation of various cell types, *CRNDE* appears as a very perspective subject of further studies.

## Materials and Methods

### Ethics statement

The ethics committee of The Maria Sklodowska-Curie Memorial Cancer Center and Institute of Oncology has approved this research.

### Cell lines and transfections

All cell line experiments were carried out on HeLa cervical carcinoma cells. The cells were grown on DMEM supplemented with 10% FBS (Life Technologies) and transfected using Lipofectamine 2000 (Life Technologies), according to the manufacturer's instructions.

### Prokaryotic and eukaryotic vectors and plasmids

Commercial bacterial vectors: pGEM-T Easy vector (Promega, Fitchburg, WI, USA); commercial mammalian expression vectors: pEGFP-N1 (Clontech), pDsRed Monomer-C1 (Clontech), pDsRed-Monomer-Golgi (Clontech), all utilized in localization experiments; Non-commercial mammalian expression vectors: mRFP-Dcp1a and pEYFP-TIA-1, both obtained from Paul Anderson, Harvard Medical School, Boston, MA, USA, pCR3-FL2 (it encodes the double FLAG tag, located upstream of the cloning site, and it was obtained from Ryszard Konopinski, Maria Sklodowska-Curie Memorial Cancer Center and Institute of Oncology, Warsaw, Poland).

### PCR experiments

All polymerase chain reactions (PCRs) were run using the AmpliTaq Gold DNA Polymerase (Life Technologies). PCR reactions consisted of the following steps: 95°C for 10 min. (hot start of the polymerase), 95°C for 30 sec. (DNA denaturation), 55°C for 30 sec. (primers annealing), 72°C for 1 min. (the PCR product elongation), 72°C for 7 min. (final elongation of the product), 4°C hold. The following steps: DNA denaturation, primers annealing and product elongation were repeated 35 times. The NTC (no template control) sample was used as a negative reference in every PCR reaction. Sequences of all PCR primers are shown in Table 1.

### Real-Time qPCR experiments

All Real-Time PCR experiments described herein were run in triplicates as singleplex reactions on the 7500 Real-Time PCR System (Life Technologies) using TaqMan Universal PCR Master Mix (Life Technologies), about 12 ng of reverse transcribed RNA per well, and the ΔΔCT method for relative quantification of gene expression, according to Life Technologies' recommendations. Expression of *CRNDE* was evaluated with the use of two personally-designed TaqMan assays (6-FAM-labelled), specifically detecting either the CRNDEP-coding transcript (FJ466686) or the other CRNDEP-non-coding transcripts (see Table 1 for details) [4, 5]. The *CRNDE* expression was normalized to a single housekeeping gene, *HGPRT* (VIC-labelled, assay id: 4326321E, Life Technologies) in reactions confirming enforced overexpression of 2xFLAG-CRNDEP in HeLa cells, where the difference in levels of the CRNDEP-coding transcript between the analyzed and control samples was very clear (see S11 Fig). On the other hand, a set of three relatively stable reference genes: *HGPRT*, *B2M* (6-FAM-labelled, assay id: Hs99999907_m1) and *GAPDH* (6-FAM-labelled, assay id: Hs99999905_m1) (all from Life Technologies) was utilized in the studies, where the differences between samples were more subtle, i.e., the shRNA-mediated knockdown of *CRNDE* (see Fig 5A).

### Cloning of the 84aas CRNDEP ORF into 3 expression vectors

There were 3 mammalian expression vectors used for cloning in this study: pEGFP-N1, pDsRed Monomer-C1 and pCR3-FL2. Considering that the first two constructs were obtained in a similar way, a general procedure will be described herein with the differences indicated in Table 2. First, PCR reactions were performed using the pQE30_CRNDE-84aas plasmid (personally made) as a template and two different sets of primers with restriction sites located to primer overhangs (see Tables 1 and 2 for details). Then, each PCR product was cloned into the pGEM-T Easy vector, followed by the cleavage of both the product and the expression vector with restriction enzymes matching the restriction sites added during PCR, ligation and transformation into the *E*. *coli* DH5α strain (see Table 2). On the contrary, the plasmid pCR3-FL2-CRNDEP was created by cleaving both the pEGFP-N1_CRNDEP plasmid and the pCR3-FL2 vector with the BamHI restriction enzyme, which allowed for subsequent subcloning of the CRNDEP insert (272 bp) without a disruption of the original reading frame. All the constructs were isolated using the Plasmid Mini kit (A&A Biotechnology, Gdynia, Poland) and sequenced twice with the use of either L388wewF or L388wewR primer and the BigDye Terminator v3.1 Cycle Sequencing Kit (Life Technologies).

**Table 2:** The most important differences between the cloning experiments performed herein.

Vector name	PCR primers (restriction sites added)	PCR template	PCR product (length)	*E*. *coli* target strain (concentration of a selective antibiotic)	Cloning verification methods
pEGFP-N1	L388BglII, L388SmaI (*BglII*, *SmaI*)	pQE30_CRNDE-84aas plasmid	6xHis-CRNDE-84aas ORF (318 bp)	DH5α (kanamycin 25 μg/ml)	*BamHI* digestion followed by agarose gel electrophoresis; DNA sequencing; Western blotting
pDsRed Monomer-C1	L388-SacI, L388-SmaIv2 (*SacI*, *SmaI*)	pQE30_CRNDE-84aas plasmid	6xHis-CRNDE-84aas ORF (319 bp)	DH5α (kanamycin 25 μg/ml)	PCR reaction with the use of RedL388F and RedL388R primers; DNA sequencing
pGEM-T Easy	various^1^	various^1^	various^1^	JM109/TOP10 (ampicillin; 100 μg/ml)	Blue/white screening of colonies on plates with IPTG and X-gal; DNA sequencing
pCR3-FL2	N/A^2^	N/A^2^	N/A^2^	DH5α (ampicillin; 100 μg/ml)	DNA sequencing

^1^) PCR products shown in this table were first cloned into the pGEM-T Easy vector and then subcloned into one of two target expression vectors listed above. This approach facilitated the cleavage of DNA inserts with restriction enzymes.

^2^) The pCR3-FL2-CRNDEP plasmid was created by subcloning the CRNDEP insert from the pEGFP-N1_CRNDEP plasmid (without PCR reactions); N/A—not applicable.

### Tricine-SDS-PAGE

Proteins were separated in gels using the method named tricine-sodium dodecyl sulfate-polyacrylamide gel electrophoresis (tricine-SDS-PAGE), suitable for separation of small proteins [42], according to the protocol by Schaegger and von Jagow [43].

A pellet of HeLa cells was suspended with a Hamilton Microsyringe (Thermo-Fisher Scientific, Waltham, MA, USA) in 8M urea and then the cells were lysed and proteins denatured in 95°C for 10–15 min. to diminish the risk of their deposition in inclusion bodies. A lysate from about 250 000 cells was loaded into a single well. After electrophoresis, gels were either stained (with silver nitrate or Coomassie Brilliant Blue R-250) or used for Western blotting.

### Antibodies

All antibodies used in the present study are characterized in Table 3.

**Table 3:** Primary and secondary antibodies used in the present study.

Type of the antibody	Reactivity (isotype)	Host	Conjugate	Working dilution (Western blot^1^ / immunofluorescence^2^ / IHC^3^)	Manufacturer
Primary / monoclonal	Anti-6xHis tag (IgG2a)	Mouse	Unlabeled	1:1000^1^	Sigma-Aldrich
Primary / monoclonal	Anti-FLAG tag (IgG1)	Mouse	HRP	1:500^1^	Sigma-Aldrich
Primary / polyclonal	Anti-GFP (IgG)	Goat	Unlabeled	1:1000^1^	Thermo-Fisher Scientific
Primary / polyclonal	Anti-CRNDEP-Epitope1 (IgG)	Rabbit	Unlabeled	1:5^1^,^4^	Abgent, Inc.
Primary / polyclonal	Anti-CRNDEP-Epitope2 (IgG)	Rabbit	Unlabeled	1:1000^1^ / 1:500^2^ / 1:800^3^	Abgent, Inc.
Primary / polyclonal	Anti-CRNDEP-Epitope3 (IgG)	Rabbit	Unlabeled	1:5^1^,^4^	Abgent, Inc.
Primary / polyclonal	Control IgG	Rabbit	Unlabeled	N/A	Sigma-Aldrich
Secondary / polyclonal	Anti-mouse IgG (H+L)	Goat	HRP	1:10000^1^	Thermo-Fisher Scientific
Secondary / polyclonal	Anti-goat IgG (H+L)	Rabbit	HRP	1:10000^1^	Thermo-Fisher Scientific
Secondary / polyclonal	Anti-rabbit IgG	Donkey	HRP	1:10000^1^	GE Healthcare (Little Chalfont, UK)
Secondary / polyclonal	Anti-rabbit IgG (H+L)	Goat	Alexa Fluor 488	1:5000^2^	Life Technologies
Secondary / polyclonal	Anti-rabbit IgG (H+L)	Goat	Alexa Fluor 647	1:300^2^	Life Technologies
Secondary / polyclonal	Anti-rabbit IgG	Goat	Biotin	1:1500^3^	Immunotech

^1^) Western Blot.

^2^) Immunofluorescence.

^3^) IHC (Immunohistochemistry).

^4^) Two of three anti-CRNDEP antibodies were not purified (Western blots were performed using rabbit sera with relatively low concentration of specific antibodies). IHC—immunohistochemistry; HRP—horseradish peroxidase; N/A—not applicable.

### Western blot studies

Initially, Western blot experiments were carried out according to the protocol by Sambrook and Russell [44]. The procedure was subsequently modified to facilitate a detection of small proteins. These modifications included: a wet protein transfer to the UltraCruz Nitrocellulose Pure Transfer Membrane, 0.22 μm (Santa Cruz Biotechnology, Inc., Dallas, TX, USA) in 1xTBE buffer on ice, 30 V, 1.5 h followed by a fixation of proteins to the membrane with 0.4% paraformaldehyde (Sigma-Aldrich, St. Louis, MO, USA) in PBS, pH 7.4, as recommended by Suzuki et al. [23]. Then, the membrane was washed with PBS, pH 7.4 (3x 10 min.), washed once with TBS-T (TBS + 1‰ Tween 20) for 10 min., blocked with the blocking buffer (5% fat-free milk in TBS-T) for 1 h at room temperature, and incubated overnight in the same buffer with the appropriate primary antibody at 4°C. Successive steps depended on a type of the primary antibody and the method of its detection (see Table 3 for details). In case of HRP-conjugated antibodies, the peroxidase activity was detected with the SuperSignal West Pico Chemiluminescent Substrate (Thermo-Fisher Scientific).

### Immunoprecipitation of the CRNDEP peptide

CRNDEP was immunoprecipitated in native conditions using total protein lysates, which were obtained by incubating 0.5 million HeLa cells for 10 min. on ice in 300 microliters of the NP-40 lysis buffer (Life Technologies), followed by their sonication using the Bioruptor Sonication System (Diagenode, Inc., Denville, NJ, USA). After centrifugation (20000 x g, 15 min., 4°C), the cell debris was discarded and the supernatant was incubated for 3.5 h, at 4°C with magnetic Dynabeads coated with Protein G (Life Technologies), bound to either the CRNDEP-specific antibody or the control IgG antibody (both developed in rabbits). The immunoprecipitation procedure was conducted according to the recommendations of the manufacturer of Dynabeads. The bound proteins were eluted by boiling the beads (95°C, 15 min.) in the 8M urea lysis buffer, pH 8.0 and the Laemmli buffer mixed 4:1 v/v.

### Cellular localization studies using fluorescence microscopy

A cellular localization of the CRNDEP peptide was evaluated with the use of constructs obtained by cloning its ORF into one of two expression vectors, pEGFP-N1 or pDsRed Monomer-C1. A subsequent transfection of the constructs into HeLa cells caused the strong, transient overexpression of two fusion proteins, 6xHis-CRNDEP-EGFP and DsRed Monomer-6xHis-CRNDEP, glowing green or red, respectively, under a fluorescence microscope.

HeLa cells growing on Lab-Tek standard coverslips (Nunc, Roskilde, Denmark) were co-transfected with two types of plasmids, either expressing cellular component-specific proteins or one of the aforementioned fusion proteins. 24 hours after transfection cells were fixed for 15 min. on ice with 4% formaldehyde in PEM buffer (80 mM PIPES, 5 mM EGTA, 2 mM MgCl_2_), washed 3x 5 min. with this buffer, quenched with 0.1 M ammonium chloride for 10 min. and permeabilised with 0.5% Triton X-100 in PEM for 5 min. Afterwards, the cells were washed 3x 5 min. with PEM buffer followed by the treatment with a mounting medium that contained DAPI. The cells were observed under one of two fluorescence microscopes, the Nikon Eclipse E-800 microscope (Nikon, Tokyo, Japan) or the Leica AF7000 microscope with the DFC350 FX camera and I3 (green) and Y5 (infrared) filer cubes (Leica, Mannheim, Germany).

Peroxisomes were immunostained with the use of SelectFX Alexa Fluor 488 Peroxisome Labeling Kit (Life Technologies). The whole procedure was conducted according to the manufacturer's recommendations.

Mitochondria were stained red with the MitoTracker Red CMXRos dye (Life Technologies), which had been added to the living cells about 20 minutes before they were fixed with formaldehyde.

### A development of custom-made polyclonal anti-CRNDEP antibodies in rabbits

Polyclonal antibodies against three different epitopes of the 84aas CRNDEP peptide were developed in rabbits (two New Zealand Rabbits per epitope) by Abgent, Inc. Each rabbit was immunized with one of the epitopes conjugated to keyhole limpet hemocyanin (KLH), which acted as a potent immunoactivator [45, 46]. Immunization of the rabbits was evaluated with ELISA tests.

### Immunohistochemical staining of the CRNDEP peptide in human tissues

Immunohistochemical stainings were performed on formalin-fixed, paraffin-embedded (FFPE) and frozen sections of various human tissues. Antigens were retrieved by heating the sections in 0.01 M citrate buffer (pH 6.0) 4 x 5 min. (at 700 W) in a microwave oven. Non-specific tissue and endogenous peroxidase reactivities were blocked with 10% BSA and 3% H_2_O_2_, respectively. Sections were incubated at 4°C overnight with the rabbit primary polyclonal anti-CRNDEP antibody, specific to the Epitope 2 (1:800, Abgent, Inc.). Biotinylated secondary goat anti-rabbit antibody (1:1500), peroxidase-conjugated streptavidin (1:500) (both from Immunotech Laboratories, Inc., Monrovia, CA, USA) and DAB were used as a detection system. Specificity of the immunohistochemical reaction was determined by using the synthetic Epitope 2 (originally utilized for rabbits' immunization during the antibody development) as a blocking peptide. The anti-CRNDEP antibody and the blocking peptide (1:3) were incubated for 2h before application. The sections were counterstained with hematoxylin and then examined under the Olympus BX41 light microscope equipped with the DP70 camera (Olympus, Shinjuku, Tokyo, Japan) or the PALM MicroBeam Laser Microdissector (Carl Zeiss AG, Oberkochen, Germany).

### Detection of endogenous CRNDEP in HeLa cells by immunofluorescence

HeLa cells were fixed on ice for 15 min. with 4% formaldehyde in PEM buffer, washed 3x for 5 min. with the same buffer, quenched with 0.1 M ammonium chloride for 10 min. and permeabilised with 0.5% Triton X-100 in PEM for 30 min. Afterwards, the cells were washed 3x for 5 min. with PEM buffer and blocked with the blocking buffer (5% powdered milk in TBS-T) for 1 hour. Next, the blocking buffer was removed and the cells were incubated overnight at 4°C with the anti-CRNDEP antibody diluted (1:500) in the blocking buffer. Later, the cells were washed 4x 5 min. with the same buffer followed by incubation (30 min., room temperature) with one of two secondary antibodies: goat anti-rabbit IgG (H+L), Alexa Fluor 488 conjugate (green signal), 1:5000 or goat anti-rabbit IgG (H+L), Alexa Fluor 647 conjugate (infrared signal), 1:300, both from Life Technologies. Then, the cells were washed 3x 5 min. with TBS-T, fixed with 4% formaldehyde in PEM for 15 min., quenched with PEM + 1 mg/ml ammonium chloride (2x 10 min.), washed with PEM (2x 5 min.) and TBS-T (2x 5 min.), treated with a mounting medium that contained DAPI, and observed under the same fluorescence microscopes that were used in cellular localization studies.

### shRNA-mediated knockdown of *CRNDE*

Herein, the *CRNDE* gene silencing was performed with the use of the pGFP-B-RS vector (OriGene Technologies, Inc., Rockville, MD, USA) harboring one of three personally-designed *CRNDE*-specific shRNAs (SH1, SH2, SH3) or a scrambled, non-silencing shRNA (SH SCR, a negative control) (see Table 1 for details). All shRNA-coding inserts were synthesized in the Institute of Biochemistry and Biophysics PAS (Warsaw, Poland) as two single-stranded, complementary 78 bp-long DNA molecules. They were later annealed into double-stranded oligonucleotides containing BamHI and HindIII sticky ends, located upstream and downstream of the shRNA-coding region, respectively. Each of these oligos was ligated with the pGFP-B-RS vector earlier cleaved with the BamHI and HindIII restriction enzymes. The obtained constructs encoded the appropriate shRNA molecule and the GFP reporter protein. In addition, they harbored the kanamycin and blasticidin resistance genes allowing for a selection of transformants / transfectants in prokaryotic and eukaryotic cells, respectively. Due to the expression of GFP, we were able to distinguish between transfected (green signal present) and non-transfected (no green signal) HeLa cells. All the constructs were sequenced twice using either the U6prom-F or SV40rev sequencing primer and the BigDye Terminator v3.1 Cycle Sequencing Kit (Life Technologies) supplemented with 5% DMSO and 40 μM dGTP. Acquisition of fluorescence images of HeLa cells was performed with the Leica AF7000 microscope equipped with the DFC350 FX camera and a motorized stage, using a 20x objective (all manufactured by Leica). By using the ImageJ application [47], the effects of *CRNDE* silencing were quantified according to the protocol described by Burgess et al. [48] for 100 knocked-down and 100 control HeLa cells. Afterwards, the obtained corrected total cell fluorescence (CTCF) values in the red channel (corresponding to the expression of CRNDEP) were subjected to a statistical analysis using the Mann-Whitney U test.

## Supporting Information

S1 Fig The Globplot2-based analysis of *CRNDE* hypothetical peptides. The results were obtained for the 84aas peptide (A), 45aas peptide (B) and 43aas peptide (C). Only the longest one was able to form a stable conformation.
(TIF)

S2 Fig A hypothetical model of the 84aas CRNDE peptide, visualized in Swiss-PdbViewer. This is the most probable 3-dimensional structure of CRNDEP, computed by the I-TASSER meta server (C-Score = -2.35, TM-Score = 0.44±0.14). The epitopes 1, 2 and 3 are marked yellow, red and green, respectively.
(TIF)

S3 Fig Computations of the 3-dimensional structure of 84aas CRNDEP. This research was made on the BioInfoBank meta server with the use of three methods (psipred, sam-t02-dssp, sam-t02-stride). All of them revealed that the peptide may form a potentially correct secondary structure.
(TIF)

S4 Fig Computations of the secondary structure and solvent accessibility for 84aas CRNDEP on the I-TASSER meta server. (TIF)

S5 Fig Predictions of antigenicity for 84aas CRNDEP. Three alternative algorithms were used, by Hopp and Woods (A) [20], Sweredoski and Baldi (B) [21], and Kolaskar and Tongaonkar (C) [22]. The higher the peaks in Fig A and B the more probable that antibodies will “see” these residues.
(TIF)

S6 Fig A search for proteins highly similar to 84aas CRNDEP made by the I-TASSER meta server. (TIF)

S7 Fig 84ass CRNDEP binding sites predicted by the I-TASSER meta server. (TIF)

S8 Fig Top 5 enzyme homologs of 84aas CRNDEP found by the I-TASSER meta server. (TIF)

S9 Fig Additional localization studies of 84aas CRNDEP in a fusion with a fluorescent tag. There was no co-localization between CRNDEP in a fusion with either EGFP (green) or DsRed Monomer (red), and the markers specific to: the Golgi apparatus (A), mitochondria (B), peroxisomes (C), and processing bodies (D) The nuclei were stained blue with DAPI.
(TIF)

S10 Fig Exemplary results of immunohistochemical staining with the use of anti-CRNDEP antibody. One can see both nuclear and cytoplasmic localizations of CRNDEP in the frozen tissue from ovarian cancer (A). Upon addition of the blocking peptide, no immunostaining is visible (B).
(TIF)

S11 Fig Real-Time qPCR results of enforced 2xFLAG-CRNDEP overexpression in HeLa cells. Green bars represent the *CRNDE* expression after transfection with the pCR3-FL2-CRNDEP plasmid. HeLa cells transfected with the same vector lacking the CRNDEP-coding ORF were used as both a negative control and a calibrator (red bars). It is worth noting that the Y axis is presented in a logarithmic scale.
(TIF)

S12 Fig The Real-Time qPCR-based analysis of expression of the CRNDEP-encoding transcript in 21 normal human tissue sets, normalized to one of four reference genes (*ACTB*, *B2M*, *GAPDH*, *HGPRT*). In this study our personally-designed TaqMan assay specific to this particular transcript was used [4, 5]. The following tissues were analyzed: 1-adrenal gland (62), 2-bone marrow (8), 3-brain, cerebellum (24), 4-brain, whole (2), 5-fetal brain (21), 6-fetal liver (63), 7-heart (10), 8-kidney (14), 9-liver (1), 10-lung, whole (3), 11-placenta (4), 12-prostate (32), 13-salivary gland (24), 14-skeletal muscle (7), 15-testis (39), 16-thymus (3), 17-thyroid gland (64), 18-trachea (?), 19-uterus (8), 20-spinal cord (49), 21-colon (5). The tissue sets numbered 1–20 came from the Human Total RNA Master Panel II (Clontech). The colonic tissue set (21) was prepared in our laboratory and was used as a calibrator. The number of samples in each set is enclosed in brackets. The Y axis is shown in a logarithmic scale.
(TIF)

S13 Fig Western blot-based verification of the specificity of our custom-made polyclonal anti-CRNDEP antibody (the PVDF membrane stained with Ponceau S being a loading control to Fig 2E). M—Spectra Multicolor Low Range Protein Ladder; 1—DsRed Monomer-6xHis-CRNDEP (340 aas, 38.5 kDa); 2—purified 14 kDa protein with the 6xHis tag, 1.4 μg (a negative control of the antibody's specificity, non-commercial); 3—6xHis-CRNDEP-EGFP (346 aas, 39.2 kDa); 4—empty; 5—EGFP (239 aas, 26.9 kDa, a negative control); 6—DsRed Monomer (232 aas, 26.2 kDa, a negative control). The purified 14 kDa protein containing the 6xHis tag in lane 2 is undetectable in these conditions.
(TIF)
