# Supplementary figures and images for: The Novel Gene CRNDE Encodes a Nuclear Peptide (CRNDEP) Which Is Overexpressed in Highly Proliferating Tissues
Source: PLoS One. 2015 May 15;10(5):e0127475. doi: 10.1371/journal.pone.0127475 (PMC4433331; doi:10.1371/journal.pone.0127475)

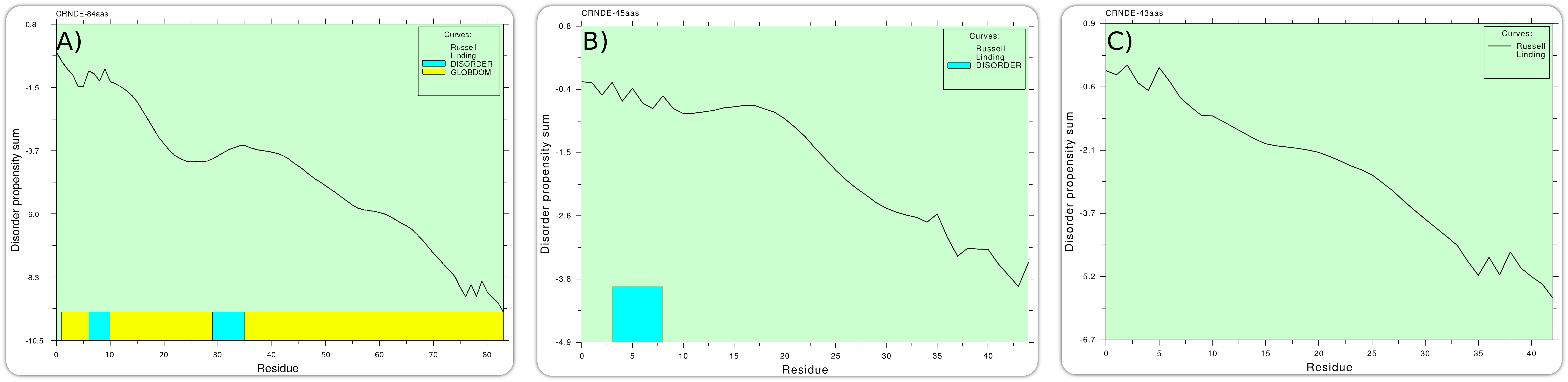

Supplement: S1 Fig — The results were obtained for the 84aas peptide (A), 45aas peptide (B) and 43aas peptide (C). Only the longest one was able to form a stable conformation. (TIF) [file pone.0127475.s001.tif]

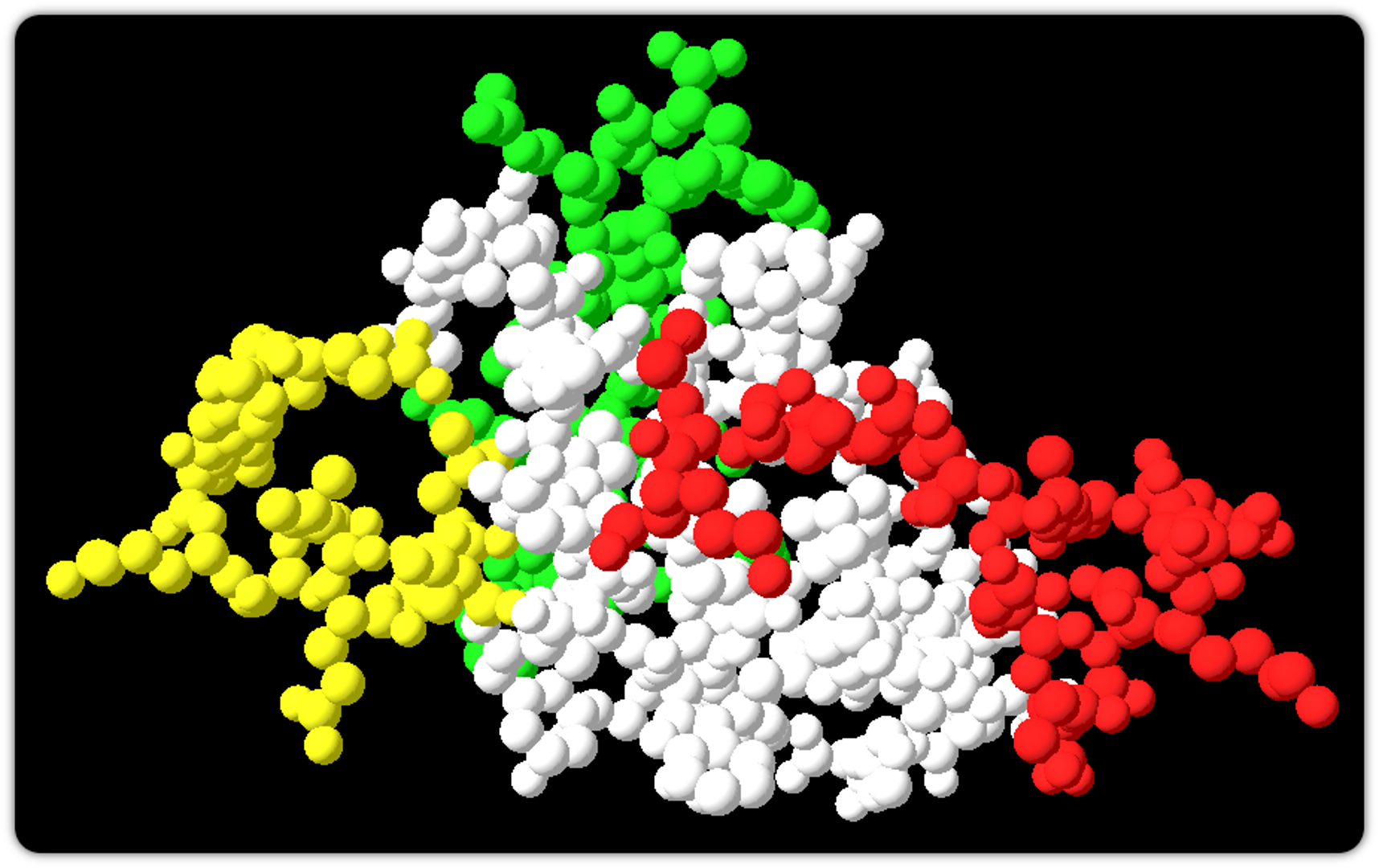

Supplement: S2 Fig — This is the most probable 3-dimensional structure of CRNDEP, computed by the I-TASSER meta server (C-Score = -2.35, TM-Score = 0.44±0.14). The epitopes 1, 2 and 3 are marked yellow, red and green, respectively. (TIF) [file pone.0127475.s002.tif]

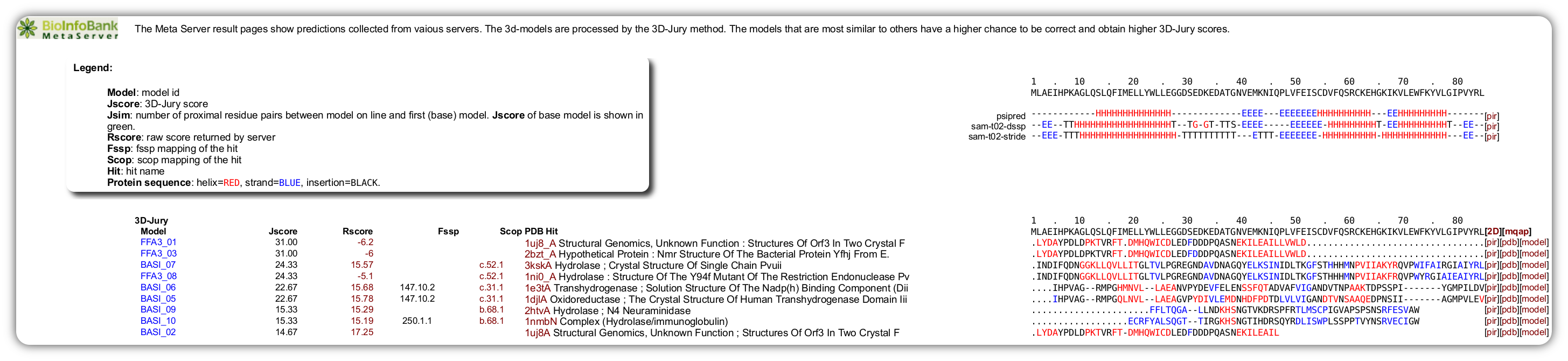

Supplement: S3 Fig — This research was made on the BioInfoBank meta server with the use of three methods (psipred, sam-t02-dssp, sam-t02-stride). All of them revealed that the peptide may form a potentially correct secondary structure. (TIF) [file pone.0127475.s003.tif]

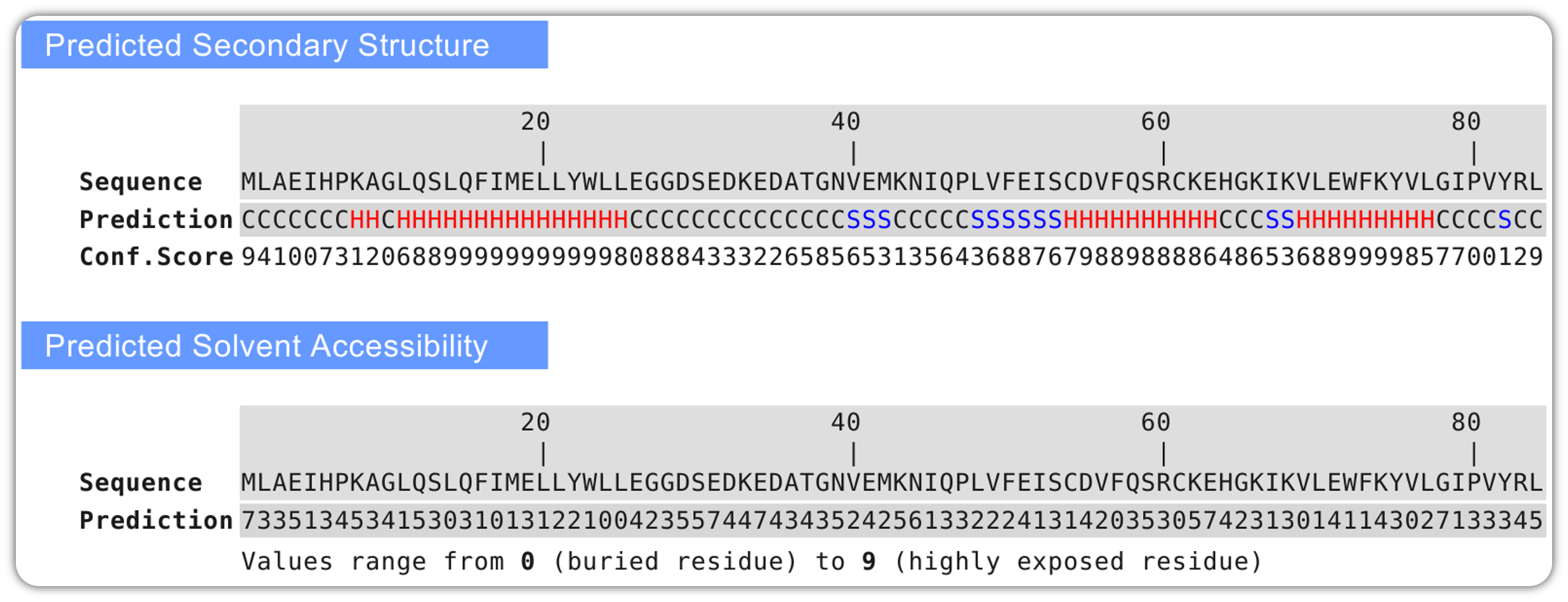

Supplement: S4 Fig — (TIF) [file pone.0127475.s004.tif]

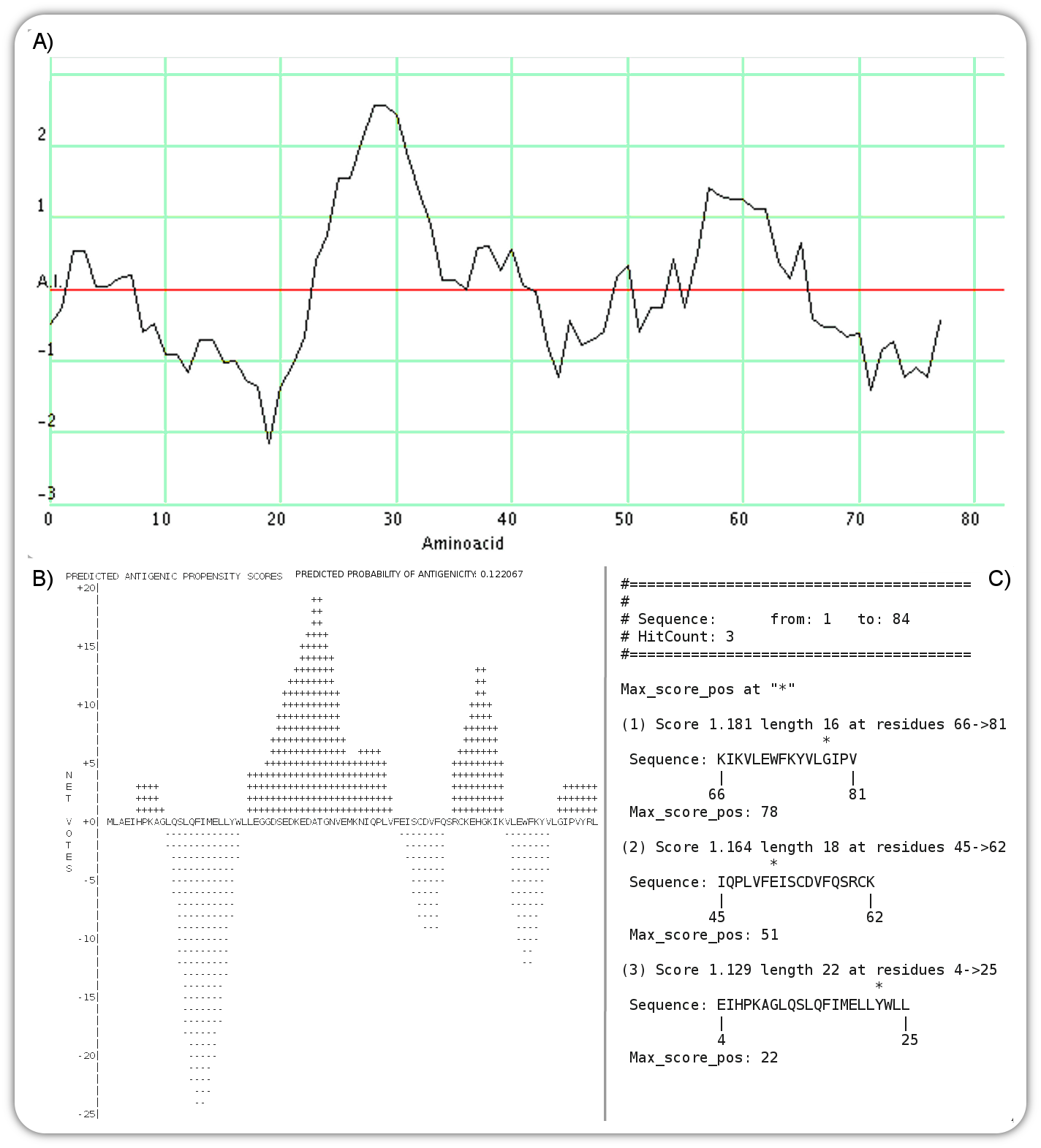

Supplement: S5 Fig — Three alternative algorithms were used, by Hopp and Woods (A) [20], Sweredoski and Baldi (B) [21], and Kolaskar and Tongaonkar (C) [22]. The higher the peaks in Fig A and B the more probable that antibodies will “see” these residues. (TIF) [file pone.0127475.s005.tif]

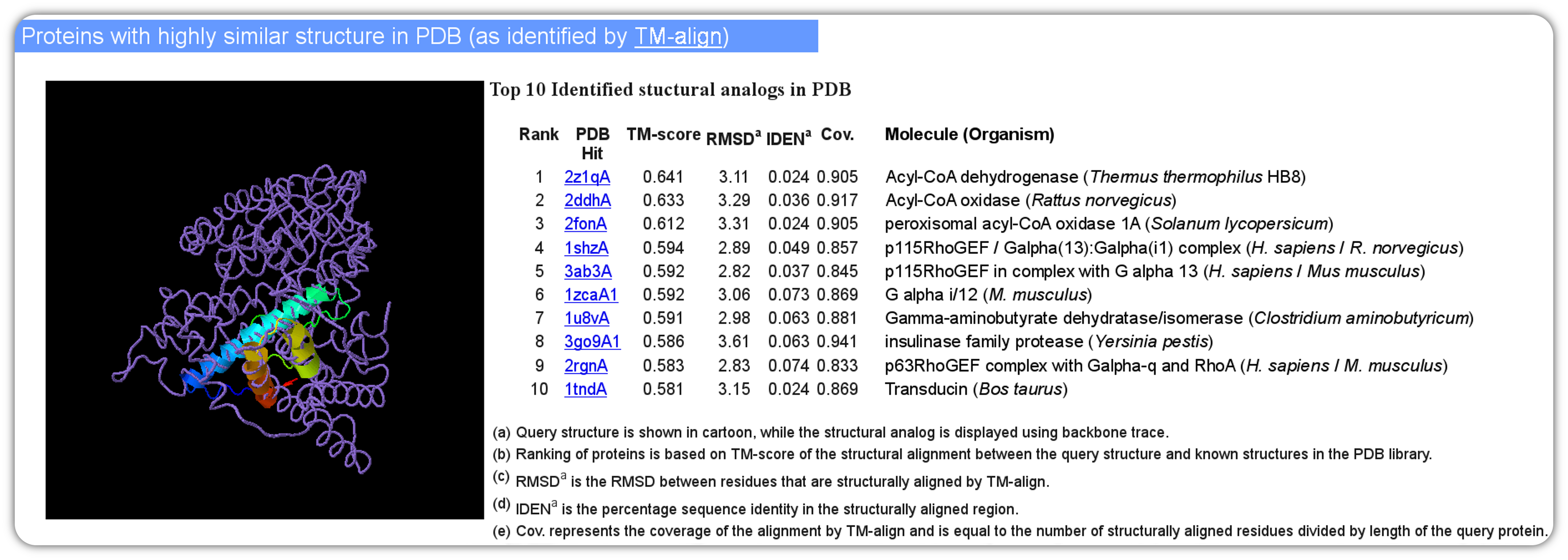

Supplement: S6 Fig — (TIF) [file pone.0127475.s006.tif]

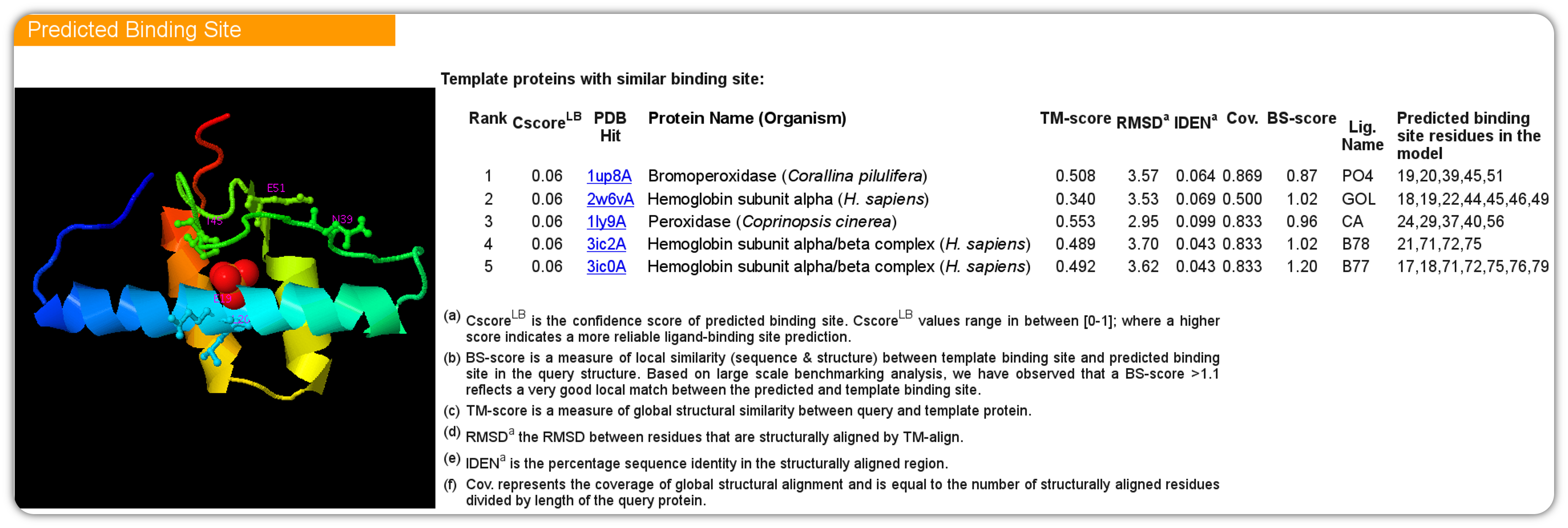

Supplement: S7 Fig — (TIF) [file pone.0127475.s007.tif]

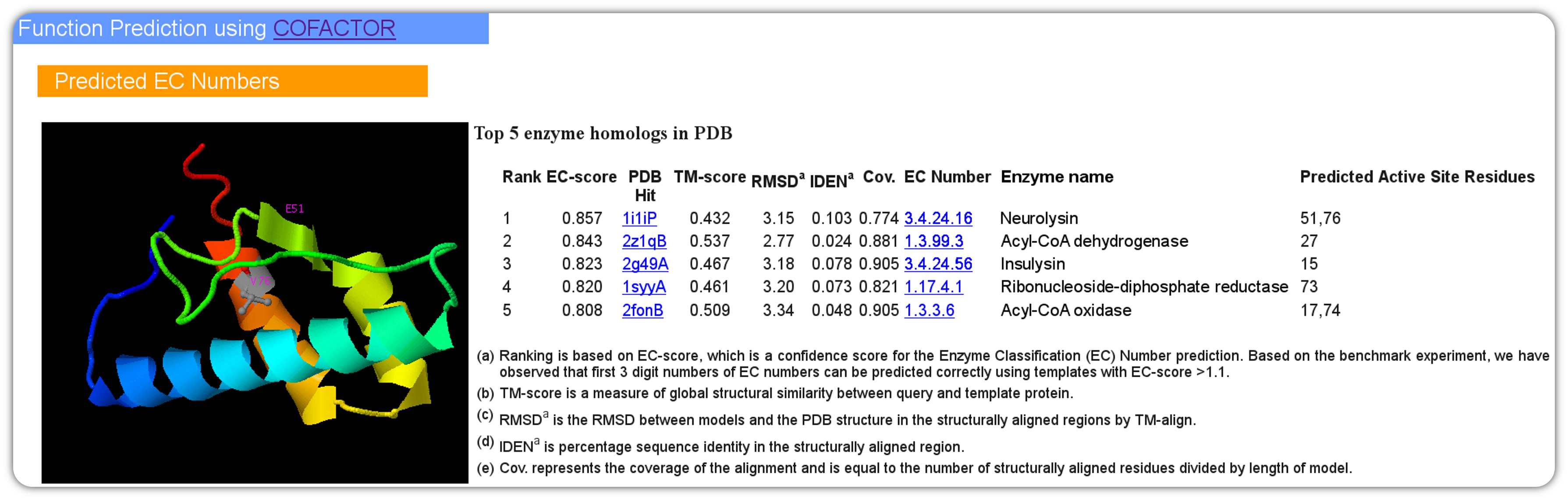

Supplement: S8 Fig — (TIF) [file pone.0127475.s008.tif]

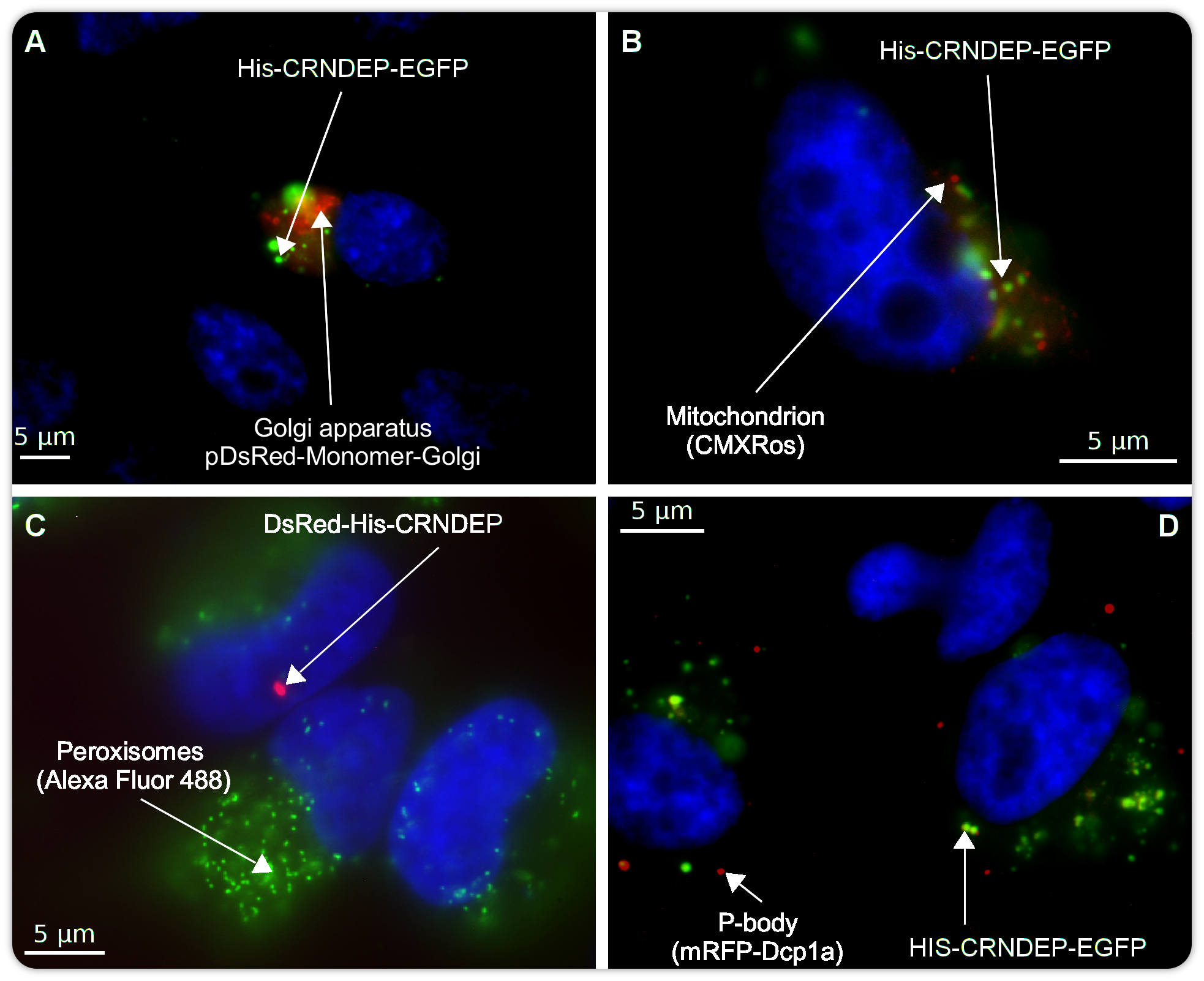

Supplement: S9 Fig — There was no co-localization between CRNDEP in a fusion with either EGFP (green) or DsRed Monomer (red), and the markers specific to: the Golgi apparatus (A), mitochondria (B), peroxisomes (C), and processing bodies (D) The nuclei were stained blue with DAPI. (TIF) [file pone.0127475.s009.tif]

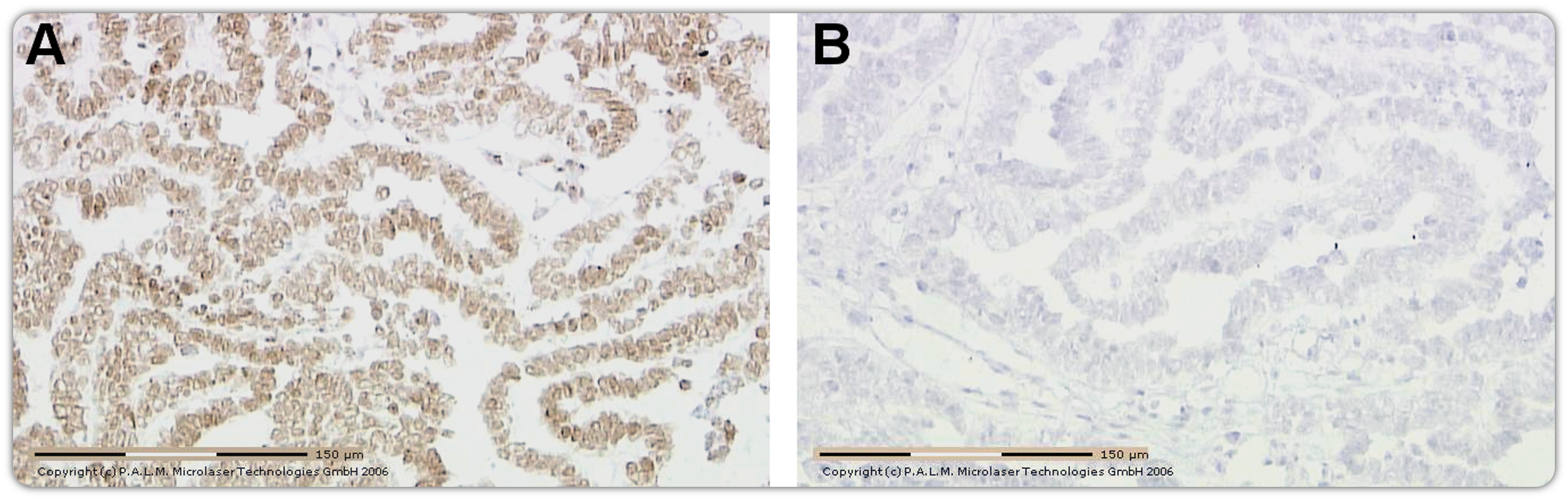

Supplement: S10 Fig — One can see both nuclear and cytoplasmic localizations of CRNDEP in the frozen tissue from ovarian cancer (A). Upon addition of the blocking peptide, no immunostaining is visible (B). (TIF) [file pone.0127475.s010.tif]

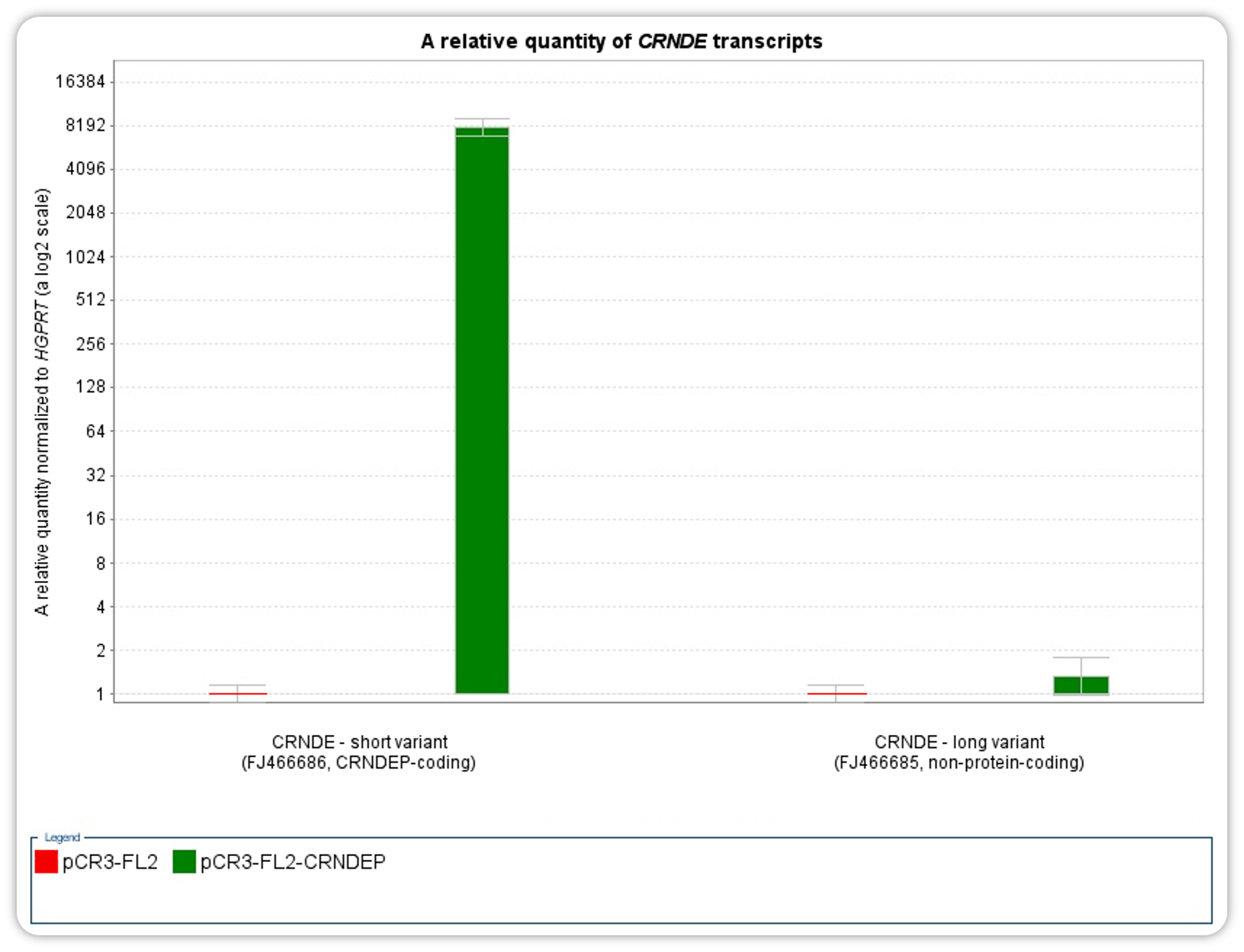

Supplement: S11 Fig — Green bars represent the CRNDE expression after transfection with the pCR3-FL2-CRNDEP plasmid. HeLa cells transfected with the same vector lacking the CRNDEP-coding ORF were used as both a negative control and a calibrator (red bars). It is worth noting that the Y axis is presented in a logarithmic scale. (TIF) [file pone.0127475.s011.tif]

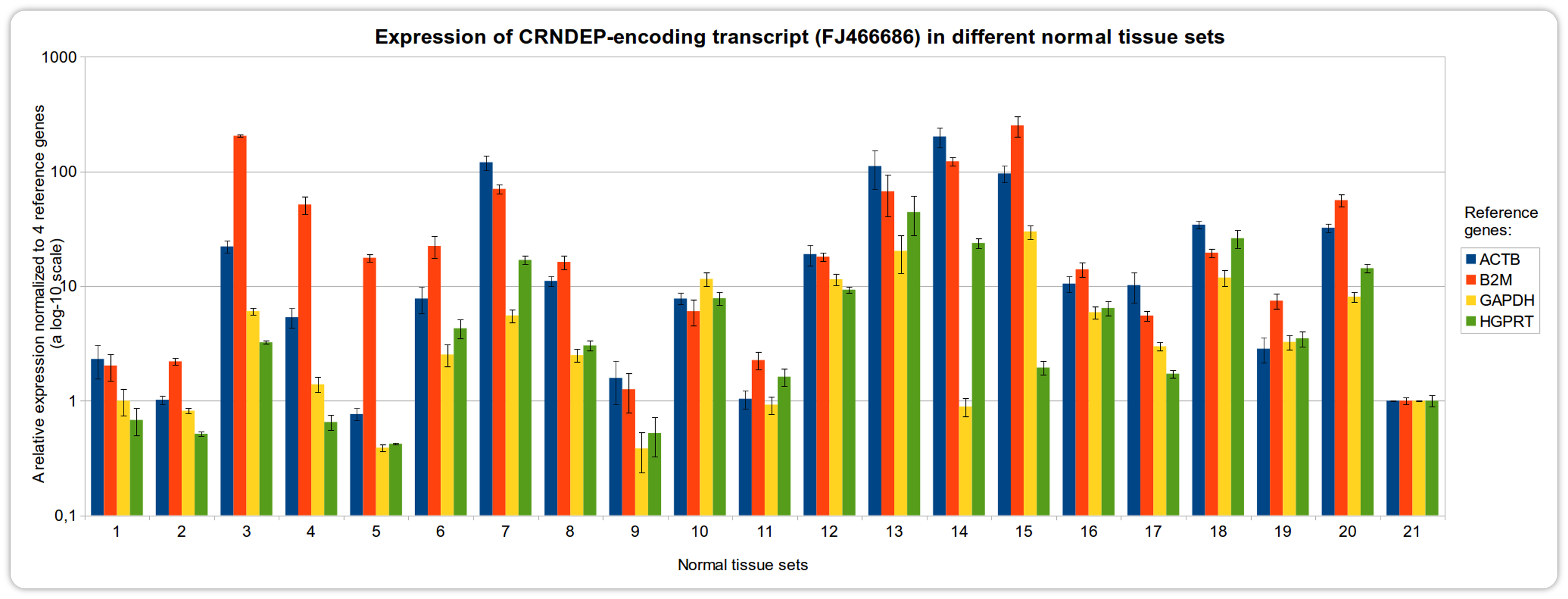

Supplement: S12 Fig — In this study our personally-designed TaqMan assay specific to this particular transcript was used [4, 5]. The following tissues were analyzed: 1-adrenal gland (62), 2-bone marrow (8), 3-brain, cerebellum (24), 4-brain, whole (2), 5-fetal brain (21), 6-fetal liver (63), 7-heart (10), 8-kidney (14), 9-liver (1), 10-lung, whole (3), 11-placenta (4), 12-prostate (32), 13-salivary gland (24), 14-skeletal muscle (7), 15-testis (39), 16-thymus (3), 17-thyroid gland (64), 18-trachea (?), 19-uterus (8), 20-spinal cord (49), 21-colon (5). The tissue sets numbered 1–20 came from the Human Total RNA Master Panel II (Clontech). The colonic tissue set (21) was prepared in our laboratory and was used as a calibrator. The number of samples in each set is enclosed in brackets. The Y axis is shown in a logarithmic scale. (TIF) [file pone.0127475.s012.tif]

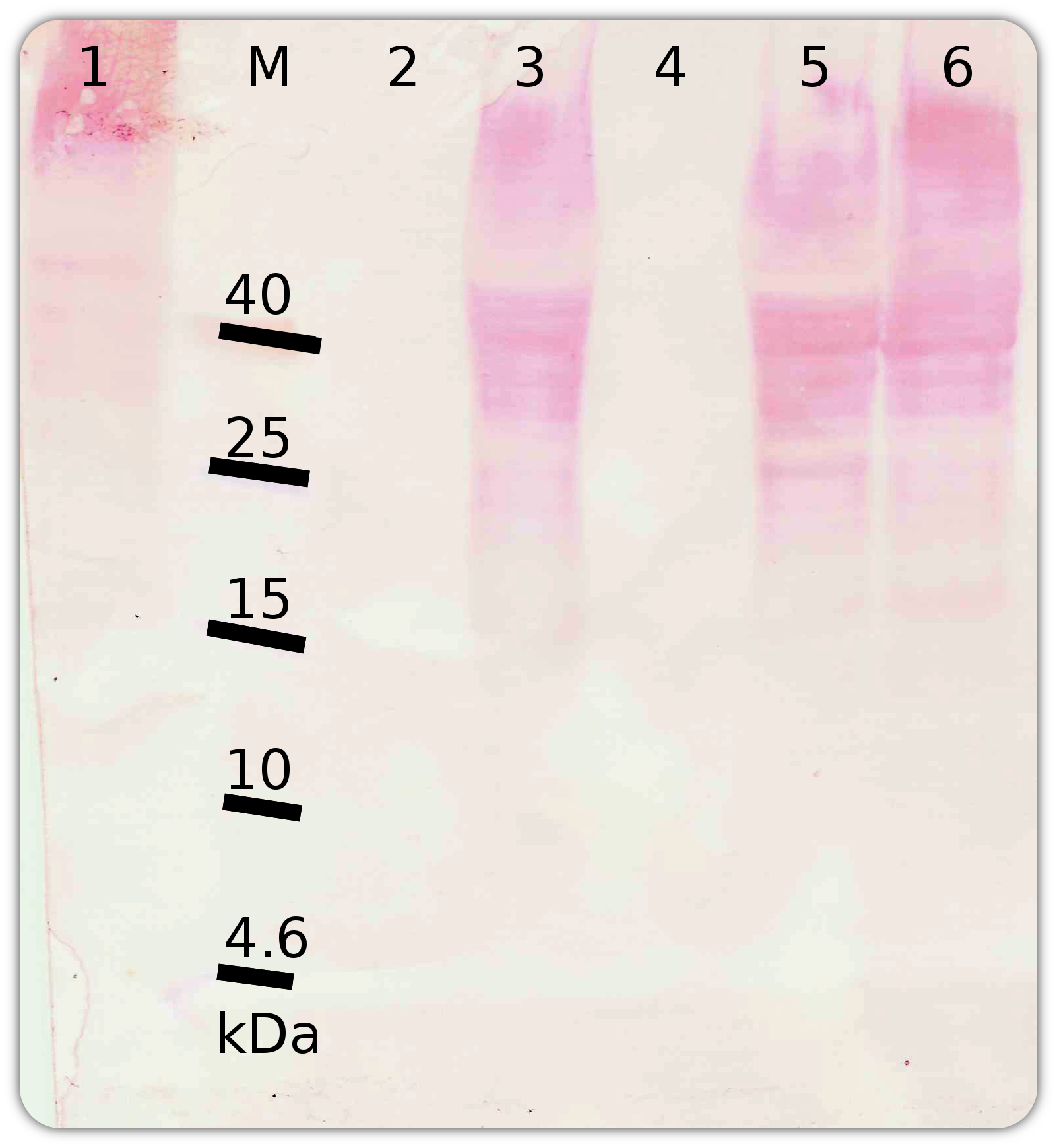

Supplement: S13 Fig — M—Spectra Multicolor Low Range Protein Ladder; 1—DsRed Monomer-6xHis-CRNDEP (340 aas, 38.5 kDa); 2—purified 14 kDa protein with the 6xHis tag, 1.4 μg (a negative control of the antibody's specificity, non-commercial); 3—6xHis-CRNDEP-EGFP (346 aas, 39.2 kDa); 4—empty; 5—EGFP (239 aas, 26.9 kDa, a negative control); 6—DsRed Monomer (232 aas, 26.2 kDa, a negative control). The purified 14 kDa protein containing the 6xHis tag in lane 2 is undetectable in these conditions. (TIF) [file pone.0127475.s013.tif]
